# Closing the gap: Nonviral TFAMoplex transfection boosted by bZIP domains compared to AAV-mediated transduction

**DOI:** 10.1016/j.omtn.2025.102526

**Published:** 2025-03-27

**Authors:** Steffen Honrath, Miguel Heussi, Lukas Beckert, David Scherer, Roderick Y.H. Lim, Michael Burger, Jean-Christophe Leroux

**Affiliations:** 1 ETH Zurich, Department of Chemistry and Applied Biosciences, Institute of Pharmaceutical Sciences, Vladimir-Prelog-Weg 3, 8093 Zurich, Switzerland; 2 Biozentrum and the Swiss Nanoscience Institute, University of Basel, Spitalstrasse 41, 4056 Basel, Switzerland

**Keywords:** MT: Delivery Strategies, gene therapy, nonviral gene delivery, protein-based transfection TFAMoplex, protein engineering, adeno-associated virus, AAV, nonviral vs viral gene delivery, bZIP domains

## Abstract

The TFAMoplex is a nanoparticulate gene delivery system based on the mitochondrial transcription factor A (TFAM) protein, which can be engineered with various functional domains to enhance plasmid DNA transfection. In this study, we aimed at improving the TFAMoplex system by incorporating basic leucine zipper (bZIP) domains, derived from the cyclic AMP (cAMP)-responsive element-binding protein (CREB), which are known to bind DNA upon dimerization. Additionally, we screened bZIP domains of other proteins (i.e., transcription regulator protein BACH1, cyclic AMP-dependent transcription factor ATF-3, and basic leucine zipper transcriptional factor ATF-like BATF) under challenging transfection conditions, identifying the bZIP domain of BACH1, bZIP_BACH1_, as particularly effective in enhancing the TFAMoplex performance, reducing the half-maximal effective concentration by more than 2-fold. We show that bZIP domains facilitate interactions with the cell membrane as single proteins and thus increase the cell association of TFAMoplexes. Finally, we compared the optimized bZIP_BACH1_-TFAMoplex to adeno-associated viruses (AAVs) regarding *in vitro* transfection efficiency and transgene expression levels. While AAVs achieved higher transfection efficiency based on the number of transfected cells, both the original and improved TFAMoplex constructs surpassed AAVs in transgene expression per cell.

## Graphical abstract

**Figure:**
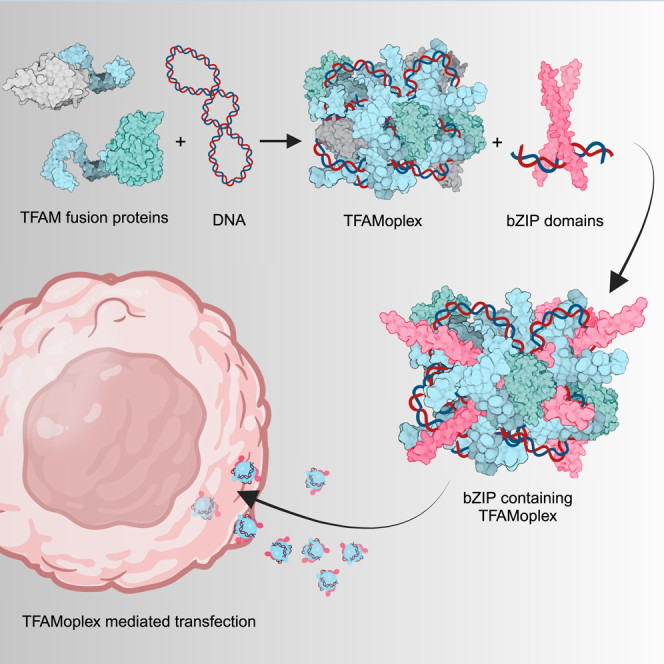

## Teaser

In this paper, Honrath and colleagues improve the TFAMoplex, a nonviral transfection system, by adding additional protein sequences, the so-called bZIP domains, to it. Finally, the transfection of these improved TFAMoplexes were compared with one of the gold standards in gene delivery, AAVs. TFAMoplexes reach higher gene expression levels.

## Introduction

With several products on the market, gene therapy enables treatment for diseases previously considered untreatable.^1^^,^^2^ Gene delivery vectors can be broadly categorized into viral and nonviral ones, each with distinct mechanisms, advantages, and limitations. Viral gene therapies rely on engineered viruses, such as adeno-associated viruses (AAVs) as vectors to transport genetic material into host cells, exploiting the natural complex viral machinery to enter cells and unpackage and deliver the genetic payload.^3^^,^^4^ This approach is known for its high transfection efficiency and tissue tropism, which is particularly advantageous for therapeutic purposes.^4^ However, the use of AAVs often carries risks such as immune responses, labor-intensive production, and limitations regarding the size of the delivered genetic material.^5^^,^^6^^,^^7^^,^^8^^,^^9^ Nonviral gene delivery methods include physical and chemical techniques such as lipoplexes, polyplexes, and protein-based systems. They are usually viewed as safer, less immunogenic, less labor-intensive, and, compared to some viruses, like e.g. lentiviruses, they cannot integrate their cargo into the host genome.^10^^,^^11^^,^^12^^,^^13^^,^^14^^,^^15^^,^^16^^,^^17^^,^^18^ Additionally, nonviral carriers are advantageous for their scalability, cost-effectiveness, and ability to deliver large genetic cargoes.^19^^,^^20^^,^^21^ However, they still suffer from reduced efficiency in comparison to viruses. Thus, the choice between viral and nonviral gene delivery depends heavily on the specific requirements of the therapeutic application, balancing efficiency and safety.

**Figure 1:**
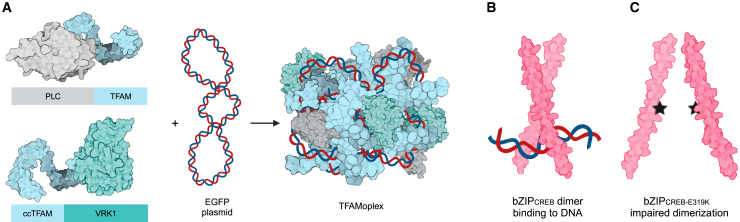
The TFAMoplex system and bZIP domains (A) The fusion proteins PLC-TFAM and ccTFAM-VRK1 associate with plasmid DNA, forming the TFAMoplex; proteins and DNA symbols are not depicted to scale. (B) The bZIP domain of CREB binds to DNA as a homodimer. (C) The E319K mutation in the dimerization site impairs the dimerization and sequence-specific DNA binding ability.

AAVs possess multifunctional proteins that form protective structures around their genetic material, facilitate endosomal escape, and enable delivery to the nucleus.^22^^,^^23^^,^^24^ These mechanisms inspired us to develop a fusion protein-based system modeled on the human mitochondrial transcription factor A (TFAM).^25^ TFAM naturally binds and compacts DNA into approximately 100-nm particles, providing a foundation for the TFAM-based transfection system, referred to as the TFAMoplex (Figure 1A).^26^^,^^27^^,^^28^^,^^29^ TFAM serves as the DNA-binding core, and additional fusion proteins provide functional versatility, mimicking the multi-purpose protein composition of viruses. An important modification to the system includes two cysteine point mutations in TFAM (ccTFAM) at the homodimerization site, which was found to boost the transfection efficiency in serum. As one of the fusion proteins, the broad-range phospholipase C (PLC) from *Listeria monocytogenes* was incorporated to promote endosomal escape.^30^ Following the TFAMoplex internalization, PLC is activated by the acidic conditions within the endosome, disrupting the membrane and allowing the release of the DNA into the cytoplasm. We also incorporated the human vaccinia-related kinase 1 (VRK1), which is intended to protect the DNA inside the cell from the protein barrier-to-autointegration factor.^25^ This is inspired by viral systems, which control the fate of their genetic cargo at every step of the transduction process.^31^^,^^32^^,^^33^^,^^34^ We demonstrated that VRK1 increases the transfection efficiency; however, its underlying mechanism remains to be clarified. The resulting TFAMoplexes, formed by combining plasmid DNA (pDNA) with an equimolar ratio of ccTFAM-VRK1 and PLC-TFAM fusion proteins achieved efficient transfection in pure serum *in vitro* at picomolar DNA concentrations and within short incubation times (<30 min).

In this study, we improved the TFAMoplex by adding basic leucine zipper (bZIP) domains. bZIP domains are responsible for sequence-specific DNA binding of transcription factors characterized by a DNA-binding basic region and a leucine-containing dimerization domain (Figures 1B and 1C).^35^^,^^36^ These proteins (e.g., CREB, BACH1) are involved in regulating genes in response to various stimuli, controlling processes such as metabolism, immune response, and cell survival.^37^^,^^38^^,^^39^^,^^40^^,^^41^ The versatility of bZIP domains arises from their ability to form homo- or heterodimers, which enables them to mediate diverse physiological responses.^35^ In the context of the TFAMoplex system, we hypothesized that an additional DNA binding site introduced by bZIP domains could lead to a higher degree of DNA complexation and thereby improve its transfection properties. In a final step, we compare for the first time head-to-head optimized TFAMoplex- and AAV-mediated transfection in serum.

## Results

### Modification of the TFAMoplex

**Figure 2:**
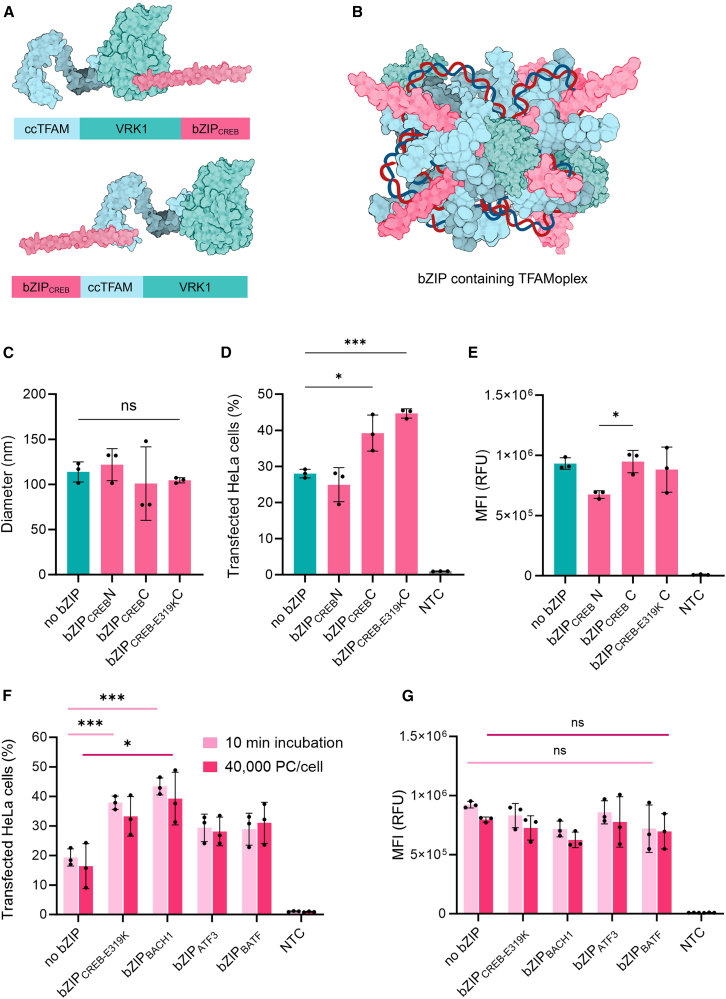
Size and transfection data of different TFAMoplex variants (A) Schematic of bZIP_CREB_ fusions to the ccTFAM-VRK1 C and N termini, resulting in the proteins bZIP_CREB_C (upper chart) and bZIP_CREB_N (lower chart). (B) Schematic representation of a TFAMoplex containing bZIP domains; protein and DNA symbols are not depicted to scale. (C) Hydrodynamic diameter of various bZIP_CREB_-containing TFAMoplexes determined by DLS in DLS buffer (100 mM KCl, 25 mM HEPES, pH 7.4). The no bZIP group indicates the standard TFAMoplex without bZIP addition. bZIP_CREB_N represents the N-terminal fusion of bZIP_CREB_. bZIP_CREB_C represents the C-terminal fusion of bZIP_CREB_. bZIP_CREB-E319K_ represents the C-terminal fusion of the E319K mutant of bZIP_CREB_. (D) Fluorescence-activated cell sorting (FACS) analysis of HeLa cells transfected with bZIP_CREB_-containing TFAMoplexes and the EGFP standard plasmid in 100% fetal bovine serum (FBS), measuring the percentage of GFP^+^ cells. The negative control (NTC) indicates untreated cells. (E) Corresponding MFI values of GFP^+^ cells from (D). (F) FACS analysis of HeLa cells transfected with TFAMoplexes containing different bZIP domains (bZIP_CREB-E319K_, bZIP_BACH1_, bZIP_ATF3_, and bZIP_BATF_) and the EGFP standard plasmid in 100% FBS under challenging conditions. Light pink bars indicate reduced incubation time (10 min instead of 30 min); dark pink bars indicate reduced total DNA amount (40,000 instead of 80,000 PC/cell). (G) Corresponding MFI values of GFP^+^ cells from (F). For (C)–(G), each dot represents the mean of an independent triplicate experiment. Data are presented as mean ± SD (*N* = 3); ∗*p* < 0.05; ∗∗*p* < 0.01; ∗∗∗*p* < 0.001.^44^

In previous experiments, the ability of TFAM to form nucleoprotein complexes together with pDNA was shown by atomic force microscopy (AFM).^42^ Here, we tested this for ccTFAM alone and in combination with wild-type TFAM (wtTFAM) using high-speed AFM (HS-AFM).^43^ We found that both conditions, ccTFAM alone and an equimolar ratio of ccTFAM and wtTFAM, formed complexes at lower concentrations than wtTFAM alone (Figure S1). This increased complexation ability might in part explain why ccTFAM-containing TFAMoplexes were able to transfect in pure fetal bovine serum (FBS).^28^ In an attempt to further improve the transfection efficiency of the TFAMoplex, we incorporated an additional DNA-binding motif in the form of a bZIP domain.^28^ Therefore, the bZIP domain of CREB, bZIP_CREB_, was N- and C-terminally fused to TFAM to identify the optimal fusion site (Figures 2A and 2B). Given that the E319K mutation in bZIP_CREB_ is known to heavily impair its ability to homodimerize, we also created a TFAMoplex incorporating the E319K mutant as a control.^45^ The DNA binding function of the TFAM domain was assessed by gel mobility shift assays (Figures S2 and S3). All proteins and their different fusion proteins were able to bind DNA, indicating that the TFAM domain in the proteins remained functional upon fusion of the bZIP domains. In the presence of PLC-TFAM and DNA, these fusion proteins formed nanoparticles (Figure 2C). The TFAMoplex hydrodynamic diameters ranged from ∼101 nm for ccTFAM-VRK1-CREB to 122 nm for CREB-ccTFAM-VRK1. However, these differences in size were not statistically significant. Polydispersity index (PDI) values also varied minimally, with the bZIP_CREB-E319K_ TFAMoplex showing the lowest PDI of 0.20 and the WT bZIP_CREB_ TFAMoplex showing the highest of 0.27, but none of the differences in the bZIP_CREB_-related groups were significant (Figure S4).

Next, we investigated the transfection efficiencies of the TFAMoplexes (Figure 2D) containing an enhanced green fluorescent protein (EGFP) reporter plasmid. HeLa cells were transfected with 80,000 plasmid copies per cell (PC/cell), which corresponds to 200 ng pDNA/mL followed by fluorescence-activated cell sorting (FACS) analysis of the transfected cells. The N-terminal bZIP_CREB_ fusion system (bZIP_CREB_N) performed similarly to the control TFAMoplex without bZIP, with transfection efficiencies of 24.9% and 28%, respectively. In contrast, the C-terminal bZIP_CREB_ fusion (bZIP_CREB_C) significantly improved the transfection efficiency, reaching 39.2%. The mean fluorescence intensity (MFI) of the transfected cells was 28% higher for the C-terminal bZIP_CREB_ fusion compared to the N-terminal fusion (Figure 2E). Surprisingly, the bZIP_CREB-E319K_ fusion (E319K), which was expected to be less efficient due to the lack of dimerization, further increased transfection efficiency to 44.7%, placing into question the role of homodimerization in complex formation and activity.

Given the diversity of bZIP proteins, we expanded the TFAMoplex by testing additional bZIP domains (Table S1).^46^ The bZIP domains were fused to the C terminus of ccTFAM-VRK1, and three variants—bZIP_BACH1_, bZIP_ATF3_, and bZIP_BATF_—were purified. Other versions (e.g., bZIP_MAFK_, bZIP_FOS_) could not be expressed in significant quantities from *Escherichia coli* and were thus not considered further. Gel mobility shift assays revealed that all bZIP proteins retained DNA in a concentration-dependent fashion (Figure S3). Interestingly, some proteins (i.e., proteins containing bZIP_CREB,_ bZIP_CREB-E319K_, and bZIP_BACH1_) showed full DNA retention already at 0.5 μM, while others (i.e., wtTFAM, ccTFAM, ccTFAM-VRK1, and the proteins containing bZIP_ATF3_ and bZIP_BATF_) exhibited only moderate DNA mobility shifts at 1 μM. After confirming the DNA-binding capacity, the transfection efficiencies were assessed.

To do this, the amount of TFAMoplex used was reduced in each well from 80,000 to 40,000 PC/cell, and, in a separate experiment, the incubation time of the TFAMoplex with the cells was shortened from 30 to 10 min while keeping the DNA amount at 80,000 PC/cell (Figure 2F). We chose these challenging conditions to better probe the differences between the samples. In both conditions, the bZIP_BACH1_ TFAMoplex outperformed the other systems, with transfection efficiencies of 39.3% in the 40,000 PC/cell experiment and 43.4% in the 10-min incubation group. In comparison, the standard TFAMoplex achieved only 16.4% and 19.3%, respectively. As an additional control, we included the bZIP_CREB-E319K_ variant, which resulted in transfection efficiencies similar to those of the bZIP_BACH1_ TFAMoplex, with 33.3% in the 40,000 PC/cell group (30 min incubation time) and 37.9% in the 10-min incubation group (80,000 PC/cell). Both bZIP_ATF3_ and bZIP_BATF_ also improved transfection compared to the standard TFAMoplex but to a lesser extent than bZIP_BACH1_ and bZIP_CREB-E319K_. For the MFI values (Figure 2G), no significant changes were observed among the different groups. Interestingly, the transfection results correlated with the gel mobility shift assays, where the best transfecting versions (bZIP_CREB_, bZIP_CREB-E319K_, and bZIP_BACH1_) showed total DNA retention in the wells with 0.5 μM protein.

**Figure 3:**
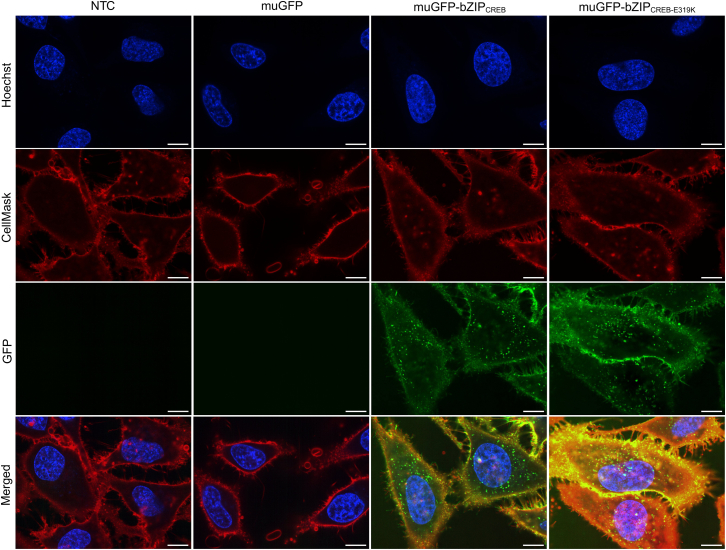
BZIP_CREB_ interaction with cell membranes HeLa cells were incubated with 500 nM of the indicated protein in 100% FBS for 30 min. Column 1: NTC with untreated cells. Column 2: treatment with monomeric ultrastable GFP (muGFP). Column 3: treatment with muGFP-bZIP_CREB_. Column 4: treatment with muGFP-bZIP_CREB-E319K_. Images are shown as single z slices in different channels. Blue: Hoechst DNA staining. Red: CellMask Deep Red. Green: muGFP signal. Merged: composite of all channels. Scale bars: 10 μm. The uncropped images are shown in Figure S5.^44^

Next, we tried to understand how bZIP proteins enhanced transfection efficiency and hypothesized that cell uptake might be increased, due to the hydrophobic and basic nature of the bZIP domains. Therefore, we fused the domains bZIP_CREB_ and bZIP_CREB-E319K_ to a green fluorescent protein (GFP).^47^ HeLa cells were incubated with these fluorescent proteins and observed by confocal microscopy. Both variants displayed a green fluorescence signal localized to the cell membrane and also within the endolysosomal system or the cytosol. Conversely, the GFP alone exhibited no fluorescence signal, indicating no cell attachment. These data suggest that the bZIP_CREB_ constructs can bind to the cellular membrane independently of their dimerization or interaction with DNA and are taken up by the cells in serum (Figure 3).

**Figure 4:**
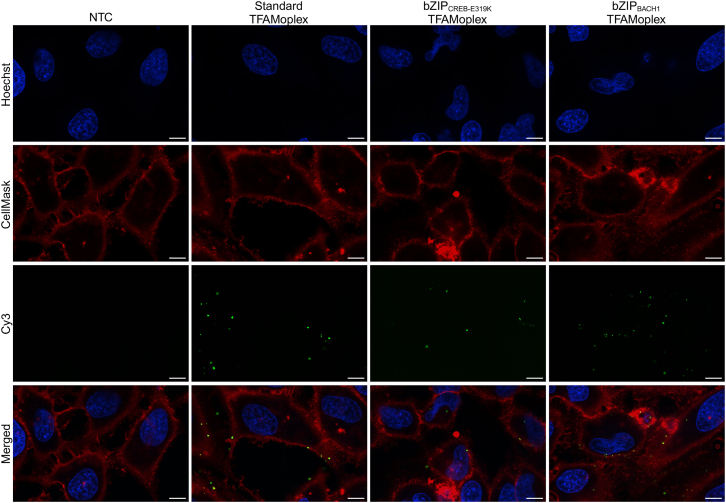
TFAMoplex association with HeLa cells in 100% FBS 30 min after addition Various TFAMoplex versions were formed with Cy3-labeled DNA and incubated with HeLa cells for 30 min, followed by confocal imaging. Column 1: NTC with untreated cells. Column 2: standard TFAMoplex. Column 3: bZIP_CREB-E319K_ TFAMoplex. Column 4: bZIP_BACH1_ TFAMoplex. Blue: Hoechst DNA staining. Red: CellMask Deep Red. Green: pseudocolored Cy3 signal of the labeled DNA. Merged: composite of all channels. Scale bars: 10 μm.^44^

In another experiment, we examined whether the bZIP-containing TFAMoplexes showed enhanced cellular association. To do this, we selected the two best-performing bZIP TFAMoplexes (bZIP_CREB-E319K_ and bZIP_BACH1_) and the standard TFAMoplex to form complexes with Cy3-labeled DNA. The labeled TFAMoplexes were incubated for 30 min with HeLa cells, and the latter were visualized by confocal microscopy. The DNA signal colocalized with the cell membrane, confirming membrane binding of the TFAMoplexes (Figure 4). The Z-projection images suggested higher association of the DNA signal to the cells for the bZIP-modified TFAMoplexes (Figure S6). These observations prompted us to quantify the cellular association of the different TFAMoplexes.

**Figure 5:**
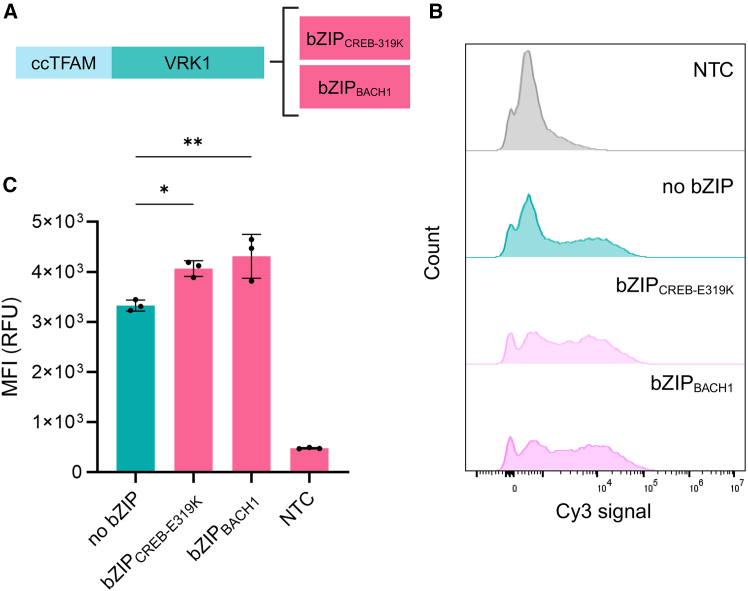
Quantification of Cy3-labeled TFAMoplexes with HeLa cells (A) Schematic illustration of the proteins used in the experiment. (B) FACS analysis of HeLa cells transfected with Cy3-labeled TFAMoplexes in 100% FBS. The Cy3 signal histogram of untreated cells (gray) is compared to cells incubated with Cy3-labeled standard TFAMoplex (teal, no bZIP), the bZIP_CREB-E319K_ TFAMoplex (light pink), and the bZIP_BACH1_ TFAMoplex (dark pink). (C) FACS analysis showing the mean Cy3 signal intensity of all cell events corresponding to (B). Each dot represents the mean of an independent triplicate experiment. Data are presented as mean ± SD (*N* = 3); ∗*p* < 0.05; ∗∗*p* < 0.01; ∗∗∗*p* < 0.001.^44^

Next, the amount of Cy3-labeled TFAMoplexes associated with the cells was quantified by flow cytometry (Figures 5A–5C). Cells incubated with the standard TFAMoplex exhibited the lowest fluorescence intensity, followed by the E319K group, with 22% higher signal and the BACH1 variant, with 30% higher signal, indicating a more pronounced cellular attachment of TFAMoplexes containing bZIPs.

### Comparison of TFAMoplex-based transfection against AAVs

**Figure 6:**
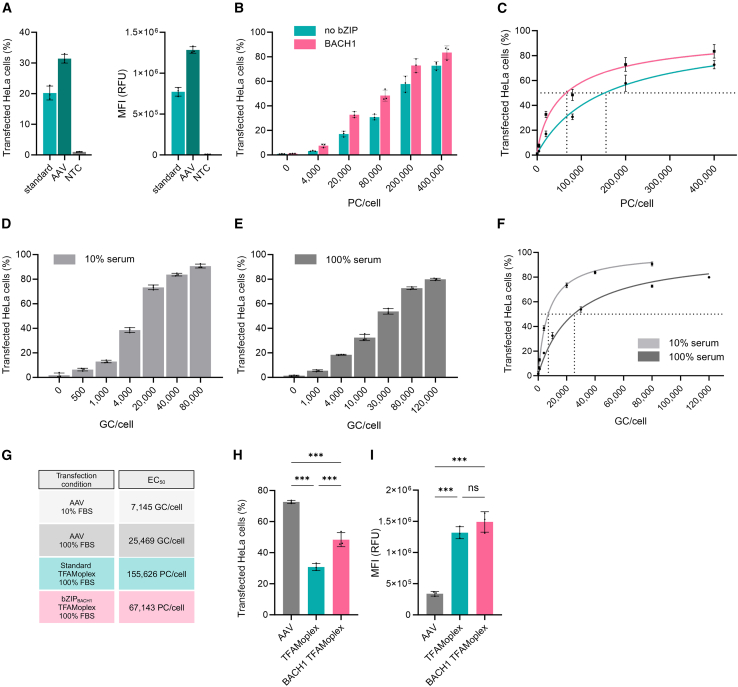
Comparison of TFAMoplex and AAV-mediated gene delivery (A) FACS analysis of HeLa cells transfected with the standard TFAMoplex and standard EGFP plasmid (light teal) or the AAV plasmid (dark teal) in 100% FBS, measuring the percentage of GFP^+^ cells. (B) FACS analysis of HeLa cells treated with the AAV plasmid and standard TFAMoplex (teal) or BACH1 TFAMoplex (pink) at varying plasmid concentrations per cell (PC/cell) in 100% FBS, measuring the percentage of GFP^+^ cells. (C) Corresponding nonlinear regression plot of transfection efficiency values for the standard TFAMoplex (teal) and BACH1 TFAMoplex (pink). (D and E) FACS analysis of HeLa cells transfected with AAVs with increasing AAV2 genome copies per cell (GC/cell) in DMEM with 10% FBS (D) or in 100% FBS (E), measuring the percentage of GFP^+^ cells. (F) Corresponding nonlinear regression plot of transduction efficiency values for AAV2 in DMEM (light gray) and 100% FBS (dark gray). (G) EC_50_ values of the different transfection agents. Both TFAMoplexes were transfected in 100% FBS. (H) FACS analysis of HeLa cells treated with AAV2 (80,000 GC/cell) and TFAMoplexes (80,000 PC/cell) in 100% FBS, comparing the standard TFAMoplex and the BACH1 TFAMoplex, measuring the percentage of GFP^+^ cells. (I) Corresponding MFI values for (G). For (A)–(F), (H), and (I), each dot represents the mean of an independent triplicate experiment. Data are presented as mean ± SD (*N* = 3); ∗*p* < 0.05; ∗∗*p* < 0.01; ∗∗∗*p* < 0.001.^44^

In previous studies, we compared TFAMoplex-based transfection primarily with lipofectamine. We reported that the TFAMoplex outperformed lipofectamine in terms of transfection efficiency.^29^ In this study, we aimed at evaluating the TFAMoplex performance against viral vectors.^28^^,^^29^ Specifically, we compared the transfection properties of two TFAMoplex variants, the standard TFAMoplex and the bZIP_BACH1_-containing version, to those of AAV serotype 2 (AAV2), which is known for efficient transduction of HeLa cells.^48^^,^^49^ To standardize the assay, the plasmid originally designed for AAV packaging was used in the TFAMoplexes (Figure S7).^50^ We compared the TFAMoplex transfection efficiency using the AAV plasmid against the standard enhanced GFP plasmid (pEGFP) used in our previous experiments.^28^^,^^29^ The AAV plasmid achieved a transduction efficiency of 31.4%, compared to 20.2% for the standard plasmid (Figure 6A). The MFI was 66% higher in the AAV plasmid, likely due to the presence of a woodchuck hepatitis virus posttranscriptional regulatory element (WPRE), which enhances transcript stability and gene expression and is absent in plasmid EGFP.^51^^,^^52^ The TFAMoplex variants were then assessed for their cytotoxicity (Figure S8). While the standard TFAMoplex showed no significant differences in cell viability at up to 1,000 ng/mL DNA, corresponding to a total TFAM concentration of 160 nM, the bZIP_BACH1_ TFAMoplex showed a decrease in viability at concentrations of 500 ng/mL and above. Although the difference was statistically significant, the values remained within an acceptable range (89.5% for 500 ng pDNA/μL and 87.5% for 1,000 ng pDNA/μL). Next, we evaluated the transfection efficiency of the two TFAMoplex variants at varying plasmid concentrations (Figure 6B). The bZIP_BACH1_ version consistently outperformed the standard TFAMoplex at all plasmid concentrations tested. The half-maximal effective concentrations (EC_50_) were extrapolated from the dose-response curves shown in Figure 6C. The bZIP_BACH1_ TFAMoplex showed an EC_50_ of 67,143 PC/cell, compared to 155,626 for the standard TFAMoplex, indicating a 2.3-fold higher efficiency. The MFI of the different TFAMoplex groups did differ only at the two highest concentrations tested, where the BACH1 exhibited slightly higher values (Figure S9).

Then, cells were transducted with AAVs under the same conditions, using cell culture medium containing both 10% and 100% FBS (Figures 6D and 6E). EGFP expression was first assessed after 24 and 48 h by fluorescence microscopy (Figure S10). Since the transfection efficacy within each system was similar between the two time points, we quantified the transgene expression only after 24 h. EC_50_ values were determined for AAVs under each condition (Figure 6F). AAVs were capable of transducting cells in pure FBS, although the required number of viral particles was higher. In 10% FBS, the EC_50_ was 7,145 genome copies per cell (GC/cell), whereas in pure FBS, it increased to 25,469 GC/cell, representing a 3.6-fold difference. The MFI of transducted cells also showed significant differences; for example, the 80,000 GC/cell group in pure FBS had only 55.5% of the MFI compared to the same GC number in 10% FBS (Figures S11A and S11B). These data clearly show that serum conditions impair the transduction levels of AAVs.

When comparing the EC_50_ values of the three transfection agents under identical conditions (AAVs, standard TFAMoplex, and bZIP_BACH1_ TFAMoplex), the bZIP_BACH1_ TFAMoplex outperformed the standard TFAMoplex, while AAVs demonstrated a 2.6-fold improvement over the bZIP_BACH1_ TFAMoplex (Figure 6G). At 80,000 PC/cell, the AAVs achieved the highest transfection rate of 72.7%, followed by the bZIP_BACH1_ TFAMoplex, with 48.4%, and the standard TFAMoplex, with 30.8% (Figure 6H).

Interestingly, despite the lower transfection efficiency, both TFAMoplex variants outperformed AAV in terms of MFI. The MFI values of the two TFAMoplex groups did not differ significantly from each other, but the MFI of the bZIP_BACH1_ TFAMoplex was 4.4-fold higher than that of AAV, suggesting that the TFAMoplexes resulted in a more efficient expression of the internalized genetic material compared to AAV (Figure 6I). This suggests that although AAVs are efficient at delivering genetic material, TFAMoplexes may allow a greater number of DNA copies to reach the nucleus in this type of experiment, leading to higher amounts of expressed protein.

## Discussion

In this study, the impact of incorporating bZIP domains into the TFAMoplexes on the transfection efficiency was investigated. The addition of bZIP proteins, particularly bZIP_CREB_ and bZIP_BACH1_, was found to significantly enhance transfection, especially under challenging conditions like low DNA concentrations and short incubation times in 100% serum. This improvement may be linked to the hydrophobic and basic nature of bZIP domains, which may promote membrane interaction, as suggested by microscopy and flow cytometry data, similar to cell-penetrating peptides.^53^ Although the higher levels of cellular attachment were observed for the bZIP-containing TFAMoplexes (22% and 30% increase vs. the standard TFAMoplex for the bZIP_E319K_ and bZIP_BACH1_ versions), these differences cannot fully account for the over 2-fold improvement in transfection efficiency observed in the bZIP groups. This discrepancy suggests that additional mechanisms such as enhanced DNA complexation (gel mobility shift data) contribute to the observed effects. Also, intracellular interactions like endosomal escape and cytosolic protein binding may contribute to the enhanced transfection properties, thereby requiring further investigation.^29^^,^^54^

Compared to other nonviral transfection agents, TFAMoplexes offer some advantages. They achieve effective transfection using lower DNA doses and shorter incubation times, even under high serum conditions. Protocols utilizing common transfection agents such as poly(ethyleneimine) (PEI) or lipofectamine typically require DNA concentrations of approximately 1 µg/mL.^55^^,^^56^^,^^57^ Incubation times for these conventional agents range from 4 to 48 h, whereas TFAMoplexes require only 10–30 min. Both our results and those reported by Wang et al. indicate that lipofectamine does not perform well in pure serum and that transfection is heavily based on particle sedimentation.^29^^,^^58^ Wang et al. additionally reported comparable *in vitro* transfection efficiencies with fluorinated dendrimers.^58^ However, this was achieved in 50% serum, at DNA concentrations of approximately 3 μg/mL and incubation times of 6 h.^58^ Overall, TFAMoplexes demonstrate superior *in vitro* efficiency relative to established nonviral gene delivery vectors.

In this work, we also directly compared the transfections mediated by the TFAMoplex to that of AAVs. While AAVs performed better in terms of the percentage of cells transducted, TFAMoplexes consistently produced significantly higher gene expression levels per cell. This suggests a difference in how these systems operate intracellularly. AAVs rely on many structural modifications to unpack their genetic cargo, which may limit the overall gene expression.^59^ In contrast, TFAMoplexes seem to bypass some of these restrictions, although the precise transfection mechanism remains to be elucidated. Our former work demonstrated that proteins fused to the TFAMoplex system can influence interactions with dynein motor proteins and nucleolar proteins, which subsequently affect transfection efficiency.^29^ It is plausible that TFAMoplexes interact more effectively with intracellular transport proteins, facilitating the delivery of a greater number of functional DNA molecules to the nucleus. However, these hypotheses require further investigation to confirm their validity.

The comparison between AAVs and TFAMoplexes underscores the trade-offs between viral and nonviral gene delivery systems. While AAVs are highly efficient, their limited cargo capacity and potential safety concerns highlight the advantages of nonviral systems like TFAMoplexes. The ability of the bZIP_BACH1_-modified TFAMoplex to maintain high gene expression levels in dividing cells, even in serum-containing conditions, suggests it may have potential for *in vivo* applications where a balance between transfection efficiency, safety, and scalability is critical.

In conclusion, TFAMoplexes and especially the integration of bZIP proteins, particularly bZIP_CREB_ or bZIP_BACH1_, represent promising systems in nonviral gene delivery. By enhancing the transfection properties, the bZIP TFAMoplex may offer an alternative to viral vectors, at least for *in vitro* and *ex vivo* applications. Modifications to the complexes such as changing the stoichiometry of the TFAMoplex components could be considered to further optimize the potency of the system.

## Materials and methods

### Materials

T4 ligase was purchased from New England Biolabs (NEB, Ipswich, MA). LB broth was obtained from LLG Labware (Meckenheim, Germany). Dithiothreitol (DTT), isopropyl-β-d-thiogalactopyranoside (IPTG), Tris-acetate-EDTA buffer (50×), and lysozyme were purchased from AppliChem GmbH (Darmstadt, Germany). Acetic acid, bovine serum albumin (BSA), ethanol, glucose, glycerol, heparin-agarose, HEPES, kanamycin sulfate, methanol, 2-mercaptoethanol, PEI (branched, average molecular weight 10,000 g/mol), potassium chloride, protease inhibitor cocktail, sodium dodecyl sulfate (SDS), and Tris-hydrochloride (Tris-HCl) were obtained from Sigma-Aldrich Chemie GmbH (Buchs, Switzerland). Bromophenol blue, Cell-Mask Deep Red, Coomassie Brilliant Blue G-250, Dulbecco’s modified Eagle’s medium (DMEM) high-glucose GlutaMAX, FastDigest Green Buffer (10×), FastDigest restriction enzymes (XbaI, NheI, XhoI, KpnI), FBS, GeneRuler DNA Ladder Mix, Hoechst stain, imidazole, Lipofectamine LP3000, Medium 199, penicillin-streptomycin (10,000 U/mL), phosphate-buffered saline (PBS; 2.7 mM KCl, 137 mM NaCl, 1.8 mM KH_2_PO_4_, 10.1 mM Na_2_HPO_4_, pH 7.4), trypsin-EDTA (0.25%), and UltraPure agarose were purchased from Thermo Fisher Scientific (Waltham, MA). GelRed DNA dye was obtained from Biotium (Hayward, CA). Ni-NTA agarose was purchased from Qiagen (Germantown, MD). Amicon Ultra 15 centrifugal filters (10,000 and 30,000 Da molecular weight cutoff [MWCO]) were purchased from Merck Millipore Ltd. (Tullagreen, Ireland). Syringe filters (0.22 μm), 24-well plates, and 96-well tissue culture plates were obtained from TPP Techno Plastic Products AG (Trasadingen, Switzerland). Protino Columns (14 mL) were purchased from Machery-Nagel (Düren, Germany). White microplates and 96-well U-shaped sterile polystyrene plates were obtained from Greiner Bio-One (Kremsmünster, Austria). ZEN0040 40 μL cuvettes and DTS1080 Disposable Folded Capillary Cells were obtained from Malvern Panalytical (Malvern, UK). Unless otherwise specified, all other chemicals were obtained from Sigma-Aldrich Chemie GmbH. Lämmli sample buffer (4×) was obtained from Bio-Rad (Hercules, CA).

### Cloning of plasmids

All cloning steps followed the manufacturer’s protocols for FastDigest restriction enzymes (Thermo Fisher Scientific) and T4 DNA ligase (NEB). DNA inserts were synthesized by GeneArt services from Thermo Fisher Scientific and Twist Bioscience (South San Francisco, CA). Both plasmid backbones and inserts were digested with the appropriate restriction enzymes, followed by ligation, transformation into *E. coli* DH5α, and plasmid isolation using the QIAprep Spin Miniprep Kit (Qiagen, Hilden, Germany).

To add the N-terminal bZIP_CREB_ sequence, the gene fragment was digested with NcoI and NheI and inserted upstream of the ccTFAM-VRK1 gene. For C-terminal modifications of ccTFAM-VRK1 proteins, an extension was added to the 5′ end of the VRK1 sequence via PCR using the primers TFAM-forward (5′-TGAGTTCAGTGCTGGCTA-3′) and VRK1-reverse (5′-ACCGGTACCAACTTTCCGTTTCTTCTTCG-3′), creating a KpnI restriction site downstream of the VRK1 gene. The PCR product was then digested with NheI and KpnI and ligated alongside the genes encoding the C-terminal extensions (e.g., bZIP_CREB_, bZIP_CREB-E319K_, bZIP_BACH1_, bZIP_ATF3_, bZIP_BATF_), which were digested with KpnI and XhoI. This double ligation process inserted the constructs into the plasmid backbone. For the construction of monomeric ultrastable GFP (muGFP) variants, the gene encoding muGFP with KpnI and XhoI restriction sites at the 5′ end was synthesized and inserted into a backbone for bacterial expression. The plasmid with these additional restriction sites was isolated, digested, and used for the insertion of bZIP_CREB_ and bZIP_CREB-E319K_ sequences. The final pDNA sequences and the UniProt accession numbers are provided in Tables S2 and S3.

### Expression and purification of proteins

Plasmids were transformed into *E. coli* BL21 pLysS cells. Bacteria were cultured at 37°C, with shaking at 250 rpm in 700 mL LB medium supplemented with 50 mg/L kanamycin and 0.2% glucose. When the optical density at 600 nm reached 0.5–0.7, protein expression was induced by adding 0.4 mM IPTG, and the culture temperature was reduced to 30°C. After 5 h, bacterial cells were harvested by centrifugation at 8,000 × *g* for 10 min at 4°C using a refrigerated centrifuge (ST16R, Thermo Scientific). The resulting pellet was stored overnight at −20°C. The next day, the pellet was resuspended in lysis buffer (1 M KCl, 1× PBS pH 7.4, 1 mM DTT, 1 mg/mL lysozyme, 1× protease inhibitor cocktail) and lysed by sonication on ice. PEI (0.1%) was added to the lysate, which was then centrifuged at 30,000 × *g* for 45 min at 4°C (Sorvall LYNX 6000 centrifuge, Thermo Scientific) to remove cell debris. The supernatant was filtered through a 0.22-μm syringe filter, supplemented with 10 mM imidazole, and loaded onto a 1-mL Ni-NTA agarose column. The column was washed with 10 column volumes (CV) of wash buffer (1 M KCl, 1× PBS pH 7.4, 1 mM DTT, 25 mM imidazole), and proteins were eluted in six 1-mL fractions using elution buffer (1 M KCl, 1× PBS pH 7.4, 1 mM DTT, 250 mM imidazole). Protein concentrations were estimated by spectrophotometry at 280 nm using a NanoPhotometer Pearl (Implen GmbH, Munich, Germany), and fractions containing protein were pooled. The pooled solution was diluted 7-fold with cold double-distilled H_2_O (ddH_2_O) to reduce the salt concentration to below 180 mM, then loaded onto a 1-mL heparin-agarose column to remove bacterial DNA. The column was washed with 5 CV of wash buffer (1× PBS pH 7.4, 1 mM DTT), and proteins were eluted using elution buffer (1× PBS, 1 M KCl pH 7.4). Buffer exchange into storage buffer (0.5× PBS pH 7.4, 10% glycerol) was performed using 30,000 MWCO Amicon Ultra Centrifugal Filters (Sigma-Aldrich, St. Louis, MO). The protein was concentrated to approximately 1 mg/mL, aliquoted, snap-frozen in liquid nitrogen, and stored at −80°C.

### HS-AFM imaging and data processing

All HS-AFM data were obtained using an HS-AFM 1.0 system (RIBM, Tsukuba, Japan). The system utilized a standard scanner operating in tapping mode. Throughout the experiments, QUANTUM-AC10-SuperSharp probes (nanotools GmbH) with a pristine tip radius of ≤2 nm were used. The probes had a nominal spring constant of 0.1 N/m, a resonant frequency close to 0.5 MHz, and a quality factor of around 2 in water. We maintained the set point amplitude (A_set_) at 80%–90% of the free cantilever oscillation amplitude (A_free_), which was adjusted between 2 and 3 nm, resulting in an imaging force of ∼45 pN.^60^^,^^61^

Prior to imaging, freshly cleaved mica surfaces were treated with 3 μL poly(l-lysine) (0.01%, m/v) for 3 min and rinsed three times with the TFAM imaging buffer (50 mM HEPES-NaOH pH 7.4, 150 mM KCl, 10 mM MgCl_2_). Different TFAM-containing formulations, stored in 0.5× PBS with 10% glycerol, were incubated with the EGFP reporter plasmid (6,100 bp) in the TFAM imaging buffer for 25 min at room temperature, yielding DNA-bp/TFAM ratios (bp/TFAM molecules) of 1, 5, 10, and 20. Subsequently, 3 μL of the mixture was deposited onto the poly(l-lysine)-treated mica, allowed to adsorb for 5 min, and rinsed three times with the TFAM imaging buffer to remove excess molecules.

All two-dimensional images captured using HS-AFM were corrected for drift and XY-plane tilt using custom Python-based software (which also converted the files into TIFF format).^62^ Further analysis of the HS-AFM images was carried out with ImageJ and cropped to a respective region of interest.

### Gel mobility shift assay

To evaluate the DNA-binding potential of various TFAM fusion proteins, 100 ng of pDNA was incubated with 0, 0.25, 0.5, or 1 μM of each TFAM variant in PBS. After a 30-min incubation at room temperature, 1 μL FastDigest Green Buffer was added to the mixture, which was immediately loaded onto a 0.8% (w/v) agarose gel for subsequent visual analysis of the band shift.

### Dynamic light scattering

To measure the size of TFAMoplexes, ccTFAM-fusion protein was mixed with PLC-TFAM at a final concentration of 0.4 μM each in dynamic light scattering (DLS) buffer (100 mM KCl and 25 mM HEPES pH 7.4), resulting in a total protein concentration of 0.8 μM. After gentle mixing, the EGFP reporter plasmid was added to achieve a final concentration of 10 ng/μL. The mixture was incubated for 10 min at room temperature, and the hydrodynamic diameter of the complexes was determined using a Zeta Sizer Pro (Malvern Instruments, Malvern, UK) based on light scattering intensity. The average peak values from three independent experiments were used to estimate the size.

### Cell culture

Chemically competent *E. coli* DH5α and BL21∗DE3 cells were obtained from Promega AG (Dübendorf, Switzerland). HeLa (ATCC CCL-2) cells were purchased from American Type Culture Collection (Manassas, VA). Cultivation of the cells was conducted in DMEM containing 10% (v/v) FBS and 1% penicillin-streptomycin. Cells were maintained and transfections were performed at 37°C and 5% CO_2_. Cells were used between passages 8 and 30. Cells were checked for mycoplasma contamination (MycoAlert PLUS Mycoplasma Detection Kit, Lonza, Basel, Switzerland) regularly.

### Transfection experiments

For the transfection procedure, 100,000 HeLa cells were seeded into 24-well plates to reach confluency by the following day. Once confluency was achieved, the cells were washed three times with warm PBS, and 500 μL FBS was added. TFAMoplexes were prepared in FBS containing 10 ng/μL of the 6.1-kbp EGFP plasmid (standard plasmid), with PLC-TFAM and ccTFAM-fusion proteins at a final concentration of 0.8 μM each, resulting in a total TFAM concentration of 1.6 μM. The mixture was gently mixed by fingertipping. For the AAV plasmid, DNA and protein concentrations were adjusted to maintain an equivalent number of plasmids while keeping the protein-to-DNA ratio constant. The TFAMoplex mixture was incubated for 30 min at room temperature, and 10 μL of the prepared mixture was added to the cells in 100% FBS. After a 30-min incubation at 37°C in 5% CO_2_, the cells were washed three times with warm PBS and incubated in DMEM containing 10% FBS for 20–24 h prior to flow cytometry analysis. For the transfection under challenging conditions, two parameters were changed in separate experiments: Either the incubation time of TFAMoplexes and cells were reduced to 10 min, or, in another experiment, only 5 μL of the prepared TFAMoplex mixture was added to the cells.

### AAV transduction

For AAV transduction, 100,000 HeLa cells were seeded into 24-well plates to reach confluency by the following day. Once confluency was achieved, the cells were washed three times with warm PBS, followed by the addition of 500 μL of either 100% FBS or DMEM supplemented with 10% FBS for transduction. AAVs were then added at varying concentrations and incubated for 30 min at 37°C in 5% CO_2_. After incubation, the cells were washed three times with warm PBS and subsequently incubated in DMEM containing 10% FBS for 20–24 h prior to flow cytometry analysis. All transduction experiments utilized AAV2 vectors containing a cytomegalovirus promoter, the EGFP gene, and a WPRE. These vectors were constructed and packaged by VectorBuilder (Vector ID: VB010000-9394npt), with detailed vector information available at vectorbuilder.com.

### Flow cytometry

For quantification of transfection efficiency and DNA-cell association, cells were analyzed by flow cytometry. The following protocol was used for both transfection analysis (20–24 h after transfection) and DNA-cell association analysis (4 h after transfection). Cells were washed with 500 μL PBS at 37°C and afterward detached using trypsin-EDTA (0.25%) diluted in PBS at a 1:4 (v/v) ratio for 5 min. The detached cells were then transferred to a 96-well U-bottom plate and centrifuged at 300 × *g* for 1 min at 4°C. After removing the supernatant, the cells were resuspended in ice-cold FACS buffer, composed of PBS (pH 7.4), 1% BSA, and 1 mM Na-EDTA. Cells were subsequently analyzed using a CytoFLEX Flow Cytometer (Beckman Coulter Life Sciences, Nyon, Switzerland), with an excitation wavelength of 488 nm and a 525/40-nm band-pass filter for transfection experiments and with an excitation wavelength of 561 nm and a 585/42-nm band-pass filter for DNA-cell association experiments. For each sample, 10,000 cells/well were collected. Transfection efficiency was evaluated based on GFP signal intensity using FlowJo software (Tree Star, Ashland, OR). Gating parameters were established for single-cell events, and the percentage of GFP^+^ cells in the negative control was set to 1%. The same gating parameters were then applied to all experimental groups. DNA-cell association was evaluated based on the mean Cy3 signal of single-cell events.

### Fluorescence microscopy imaging

For evaluating the EGFP signal of transfected cells microscopically, cells were washed three times with 500 μL 37°C PBS, followed by imaging in warm Live Cell imaging solution at 10× magnification by using a Leica DMi6000 Inverted Fluorescence Microscope (Leica Microsystems, Wetzlar, Germany). The expression was visualized with the fluorescence channel (excitation filter of 460–500 nm and an emission filter of 512–442 nm).

### Confocal microscopy imaging

For assessing the membrane binding of bZIP_CREB_, the proteins muGFP-bZIP_CREB_, muGFP-bZIP_CREB-E319K_, and muGFP were added to HeLa cells cultured in 8-well ibidi (ibidi, Fitchburg, WI) glass slides at a final concentration of 500 nM in 100% FBS following an incubation period of 30 min in the incubator at 37°C, 5% CO_2_. Afterward, the cells were washed three times with PBS at 37°C and then incubated with microscopy staining buffer (Hoechst stain (0.5% v/v) and CellMask Deep Red (0.25% v/v) in Medium 199) for 30 min in the incubator. Afterward, cells were washed with warm PBS and subsequently imaged in Medium 199 using a Nikon Spinning Disk SoRa microscope (Nikon, Tokyo, Japan). Excitation wavelengths of 405, 488, 515, and 647 nm and corresponding emission filters of 447, 525, 600, and 708 nm were used.

For evaluating the cell association of various TFAMoplexes, TFAMoplexes (standard, bZIP_CREB-E319K_ and bZIP_BACH1_ versions) were prepared using Cy3-labeled DNA (Mirus Bio, Madison, WI) and applied to HeLa cells grown in 8-well ibidi glass slides. After a 30-min incubation period at 37°C, 5% CO_2_, cells were washed and stained using the same protocol as described above and subsequently imaged in Medium 199 using a Nikon Spinning Disk SoRa microscope. Excitation wavelengths of 405, 515, and 647 nm and corresponding emission filters of 447, 600, and 708 nm were used.

### Cell viability assay

Cytotoxicity was assessed using the CellTiter 96 AQueous One Solution Cell Proliferation Assay (Promega). HeLa cells were seeded into 96-well tissue culture plates at a density of 5,000 cells/well and cultured in DMEM. The following day, the cells were washed three times with 100 μL PBS at 37°C and subsequently incubated with 100 μL warm FBS. TFAMoplexes were then added at varying concentrations, with control groups including proteins and DNA alone at concentrations equivalent to those in the 1,000-ng/mL TFAMoplex formulation. A 2% SDS solution was used as a positive control for cytotoxicity. Additionally, AAVs were added at the highest concentration used in the transduction experiments (150,000 GC/cell). The plates were incubated for 20–24 h at 37°C in 5% CO_2_, after which the cells were washed three times with warm PBS. The assay reagent was then added, and the plates were incubated for 1 h at 37°C in 5% CO_2_. Absorbance was measured at 490 nm using a plate reader (Spark, Tecan Trading AG, Männedorf, Switzerland).

### SDS-PAGE

To analyze the size and purity of the expressed constructs, 4 μg protein were mixed with Laemmli buffer (Bio-Rad, Hercules, CA) and boiled at 95°C for 5 min. After cooling, the samples were briefly centrifuged, and 4 μg protein were loaded per well onto precast 12-well gels (Bio-Rad). The gels were run at 100 V for 90 min in SDS-PAGE buffer (25 mM Tris-HCl, 200 mM glycine, 0.1% (v/v) SDS pH 8). Following electrophoresis, the gel was stained with Coomassie staining solution (0.1% (w/v) Coomassie blue, 10% (v/v) acetic acid, 30% (v/v) methanol in ddH_2_O) for 30 min and then incubated in destaining solution (10% (v/v) acetic acid, 30% (v/v) methanol in ddH_2_O) for 1 h. The destained gels were imaged using a ChemiDoc system (Bio-Rad).

### Statistical analysis

Statistical analysis of all transfection, cellular attachment, and DLS data shown in Figures 2, 4, and 6 was performed using GraphPad Prism version 10 (GraphPad Software, San Diego, CA). Data are presented as the mean ± standard deviation, based on at least three independent experiments. One-way ANOVA followed by Tukey’s multiple comparisons test was used to assess statistical significance.

## Data availability

All data supporting the findings of this study are available in the supplemental information. The corresponding raw data can be found in the ETH Research Collection.

## Acknowledgments

The authors gratefully acknowledge the support of the Scientific Center for Optical and Electron Microscopy (ScopeM) at 10.13039/501100003006ETH Zurich, especially Joachim Hehl, for their support with the confocal microscopy studies. The authors thank the group of Professor Yohei Yamauchi, especially Roger Meier and Marcel Brasser, for the introduction into the use of their infrastructure. The authors thank Helena Braet (Drug Formulation and Delivery, ETH Zurich, Switzerland) for proofreading the manuscript. This project has received funding from the 10.13039/501100000781European Research Council under the European Union’s Horizon 2020 research and innovation program (grant agreement no. 884505). R.Y.H.L. is supported by the Schweizerischer Nationalfonds zur Förderung der Wissenschaftlichen Forschung (10.13039/501100001711Swiss National Science Foundation, grant no. 310030_201062). L.B. is supported by a 10.13039/100011963Swiss Nanoscience Institute Ph.D. fellowship.

## Author contributions

S.H. designed and conducted experiments, purified proteins, analyzed, and interpreted the data, and wrote the manuscript. M.H. conducted the bZIP-related experiments, purified proteins, and analyzed and interpreted the data. L.B. designed and conducted the HS-AFM experiments and analyzed and interpreted the data. D.S. conducted the confocal microscopy experiments and purified proteins. R.Y.H.L. supervised the HS-AFM experiments and revised the manuscript. M.B. conceived the bZIP implementation strategy, supervised the project, designed the experiments, interpreted the data, and reviewed the manuscript. J.-C.L. supervised the project, interpreted the data, and reviewed the manuscript.

## Declaration of interests

The authors declare no competing interests.

## Declaration of generative AI and AI-assisted technologies in the writing process

During the preparation of this work the author(s) used ChatGPT-4o (OpenAI, San Francisco, CA) to improve readability and language. After using this tool, the authors reviewed and edited the content as needed and take full responsibility for the content of the publication.

## Supplemental information

Document S1. Tables S1–S3 and Figures S1–S11

Document S2. Article plus supplemental information
