# Supplementary material for: Closing the gap: Nonviral TFAMoplex transfection boosted by bZIP domains compared to AAV-mediated transduction
Source: Mol Ther Nucleic Acids. 2025 Mar 27;36(2):102526. doi: 10.1016/j.omtn.2025.102526 (PMC12018551; doi:10.1016/j.omtn.2025.102526)
Supplement: Document S1. Tables S1–S3 and Figures S1–S11 [file mmc1.pdf]

OMTN, Volume *36*

Supplemental information

Closing the gap: Nonviral TFAMoplex transfection boosted by bZIP domains compared to AAV-mediated transduction

Steffen Honrath, Miguel Heussi, Lukas Beckert, David Scherer, Roderick Y.H. Lim, Michael Burger, and Jean-Christophe Leroux

Table S1 – Net charge, size, and expressability as ccTFAM-VRK1-fusion protein of bZIP domains of different human proteins that were chosen for this study based on Uniprot entries containing bZIP domains.

bZIP domain	Net charge	Size (kDa)	Purifiable as ccTV-fusion protein
**CREB**	**+8**	**7.1**	**Yes**
**CREB E319K**	**+10**	**7.1**	**Yes**
CEBPA	+11	8.8	Low yield
CEBPG	+8	9.3	Low yield
**ATF3**	**+6**	**8.9**	**Yes**
**BATF**	**+1**	**9.0**	**Yes**
JunB	+11	8.6	Low yield
FOS	+1	8.8	Low yield
**BACH1**	**+5**	**9.1**	**Yes**
MafF	+10	17.2	Low yield
MafG	+12	17.8	Low yield
MafK	+11	17.4	Low yield

Table S2 – Gene sequences of all constructs used in this study.

**wtTFAM gene sequence**<br>ATGGGCAGCAGCCATCATCATCATCATCACAGCAGCGGCCTGGTGCCGCGCGGC<br>AGCCATATGGCTAGCATGACTGGTGGACAGCAAATGGGTCGCGgatccATGTCATC<br>TGTCTTGGCAAGTTGTCCAAAGAAACCTGTAAGTTCTTACCTTCGATTTTCTAAAG<br>AACAACTACCCATATTTAAAGCTCAGAACCCAGATGCAAAAACTACAGAACTAATTA<br>GAAGAATTGCCCAGCGTTGGAGGGAACTTCCTGATTCAAAGAAAAAAATATATCAA<br>GATGCTTATAGGGCGGAGTGGCAGGTATATAAAGAAGAGATAAGCAGATTTAAAGA<br>ACAGCTAACTCCAAGTCAGATTATGTCTTTGGAAAAAGAAATCATGGACAAACATT<br>TAAAAAGGAAAGCTATGACAAAAAAAAAAAGAGTTAACACTGCTTGGAAAACCAAAA<br>AGACCTCGTTCAGCTTATAACGTTTATGTAGCTGAAAGATTCCAAGAAGCTAAGGG<br>TGATTCACCGCAGGAAAAGCTGAAGACTGTAAAGGAAAACTGGAAAAATCTGTCT<br>GACTCTGAAAAGGAATTATATATTCAGCATGCTAAAGAGGACGAAACTCGTTATCAT<br>AATGAAATGAAGTCTTGGGAAGAACAAATGATTGAAGTTGGACGAAAGGATCTTC<br>TACGTCGCACAATAAAGAAACAACGAAAATATGGTGCTGAGGAGTGTTAA
**ccTFAM gene sequence**<br>ATGGGCAGCAGCCATCATCATCATCATCACAGCAGCGGCCTGGTGCCGCGCGGC<br>AGCCATATGGCTAGCATGACTGGTGGACAGCAAATGGGTCGCGgatccATGTCATC<br>TGTCTTGGCAAGTTGTCCAAAGAAACCTGTAAGTTCTTACCTTCGATTTTCTAAAG<br>AACAACTACCCATATTTAAAGCTCAGAACCCAGATGCAAAAACTACAGAACTAATTA<br>GAAGAATTGCCCAGCGTTGGAGGGAACTTCCTGATTCAAAGAAAAAAATATATCAA<br>GATGCTTATAGGTGTGAGTGGCAGTGTTATAAAGAAGAGATAAGCAGATTTAAAGA<br>ACAGCTAACTCCAAGTCAGATTATGTCTTTGGAAAAAGAAATCATGGACAAACATT<br>TAAAAAGGAAAGCTATGACAAAAAAAAAAAGAGTTAACACTGCTTGGAAAACCAAAA<br>AGACCTCGTTCAGCTTATAACGTTTATGTAGCTGAAAGATTCCAAGAAGCTAAGGG<br>TGATTCACCGCAGGAAAAGCTGAAGACTGTAAAGGAAAACTGGAAAAATCTGTCT<br>GACTCTGAAAAGGAATTATATATTCAGCATGCTAAAGAGGACGAAACTCGTTATCAT<br>AATGAAATGAAGTCTTGGGAAGAACAAATGATTGAAGTTGGACGAAAGGATCTTC<br>TACGTCGCACAATAAAGAAACAACGAAAATATGGTGCTGAGGAGTGT
**ccTFAM-VRK1 gene sequence**<br>ATGGGGAGTTCACACCATCATCACCACCACGGATCTGGTAGTATGAGTTCAGTGC<br>TGGCTAGCTGTCCGAAAAAACCAGTCTCTTCATATCTGCGTTTTTCAAAAGAGCA<br>GTTGCCAATCTTTAAGGCCCAAAATCCAGATGCGAAAACAACTGAGCTGATTAGA<br>CGCATAGCGCAACGGTGGAGAGAACTGCCGGACTCCAAGAAGAAGATTTATCAG<br>GACGCGTATCGCTGTGAGTGGCAATGCTATAAAGAAGAAATATCGCGTTTCAAAG<br>AACAGCTGACCCCTAGTCAGATTATGTCCCTTGAGAAAGAAATCATGGATAAACAC<br>CTGAAACGAAAAGCAATGACCAAGAAAAAAGAATTAACCTTACTGGGAAAACCAA<br>AGCGGCCGCGCAGTGCATACAATGTTTATGTGGCTGAACGGTTTCAAGAGGCAA<br>AAGGCGATTCTCCTCAGGAGAAACTGAAAACGGTTAAAGAAAATTGGAAGAACCT<br>CTCCGATTCAGAGAAGGAACTGTATATCCAGCACGCTAAAGAGGATGAAACAAGA<br>TATCATAACGAAATGAAATCCTGGGAGGAGCAGATGATTGAGGTAGGTCGGAAAG<br>ACCTTCTACGTCGCACTATTAAAAAACAGCGCAAATACGGTGCTGAAGAATGCAG<br>TGGGGGTAGCTCCGGCCGTGGATCCATGCCCCGTGTGAAGGCGGCGCAGGCTG<br>GACGGCAGTCTTCAGCGAAGCGTCACCTCGCGGAGCAGTTTGCAGTTGGAGAA<br>ATTATCACTGATATGGCTAAAAAGGAGTGGAAAGTGGGACTGCCTATTGGACAAG<br>GTGGATTTGGTTGTATCTATCTGGCCGACATGAACTCTTCGGAATCTGTGGGCTC

AGATGCTCCCTGTGTAGTCAAAGTAGAACCTTCAGATAATGGGCCGCTGTTTACT
GAACTGAAATTTTATCAAAGGGCTGCTAAACCTGAACAGATACAAAAATGGATACG
GACTCGGAAATTGAAATATCTCGGCGTACCAAAATATTGGGGTAGCGGACTTCATG
ATAAAAATGGGAAATCGTATCGTTTTATGATAATGGACCGGTTCGGCTCGGACTTA
CAAAAAATTTACGAGGCGAACGCCAAACGGTTTAGCCGCAAGACTGTATTACAGC
TGAGCTTGCGCATTCTGGATATTCTTGAGTATATCCACGAACATGAATATGTTCATG
GTGATATTAAGGCAAGCAATTTATTATTGAACTATAAGAACCCGGATCAGGTATATTT
GGTGGATTACGGTCTGGCATACCGCTACTGCCCGGAGGGAGTACACAAAGAGTA
TAAGGAAGACCCAAAACGGTGTCATGACGGAACCATCGAATTTACCTCGATAGAC
GCACATAACGGTGTCGCGCCCTCACGTCGTGGAGACCTGGAAATCCTGGGATAT
TGCATGATTCAGTGGCTGACGGGACACCTTCCGTGGGAGGATAATCTGAAAGATC
CTAAGTATGTGCGAGACAGTAAGATTAGATACAGGGAAAATATAGCCAGCCTGATG
GATAAATGCTTTCCAGAAAAGAACAAACCGGGAGAAATCGCTAAATATATGGAGAC
TGTCAAACTTTTGGATTACACCGAGAAACCGCTGTATGAAAACCTCCGCGATATTT
TACTACAGGGCCTGAAAGCCATTGGCAGTAAAGATGATGGCAAGTTAGACCTGTC
AGTGGTTGAAAACGGGGGTCTTAAAGCAAAGACAATTACGAAAAAACGAAAGAAA
GAGATTGAAGAATCAAAAGAACCAGGCGTTGAAGATACTGAATGGAGCAATACAC
AGACAGAAGAGGCTATCCAGACGCGTTCCAGAACCCGCAAACGTGTTCAGAAGA
GCGGTCCGAAGAAGAAACGGAAAGTATAA

ccTFAM-VRK1-bZIP$_{CREB}$ gene sequence

ATGGGGAGtTCACACCATCATCACCACCAcGGATCTGGTAGTATGAGTTCAGTGCT
GGCTAGCTGTCCGAAAAAACCAGTCTCTTCATATCTGCGTTTTTCAAAAGAGCAG
TTGCCAATcTTTAAgGCCCAAAATCCaGATGCGAAAACAACTGAgCTGATTAGACGC
ATAGCGCAACGGTGGAGAGAACTGCCGGACtccAAGAAgAAGATtTATCAGGACGC
gTATCGCTGTGAGTGGCAATGCTATAAAGAAGAAATATCGCGTTTCAAAGAACAGC
TGACCCCTAGTCAGATTATGTCCCTTGAGAAAGAAATCATGGATAAACACCTGAAA
CGAAAAGCAATGACCAAGAAAAAAGAATTAACCTTACTGGGAAAACCAAAGCGGC
CGCGCAGTGCATACAATGTTTATGTGGCTGAACGGTTTCAAGAGGCAAAAGGCG
ATTCTCCTCAGGAGAAACTGAAAACGGTTAAAGAAAATTGGAAGAACCTCTCCGA
TTCAGAGAAGGAACTGTATATCCAGCACGCTAAAGAgGATGAAACAAGATATCATA
ACGAAATGAAATCCTGGGAGGAGCAGATGATTGAGGTAGGTCGGAAAGAcCTTCT
ACGTCGCACTATTAAAAAACAGCGCAAATACGGTGCTGAAGAATGCAGTGGGGGT
AGCTCCGGCCGTGGATCCATGCCCCGTGTGAAGGCGGCGCAGGCTGGACGGC
AGTCTTCAGCGAAGcgtCACCTCGCGGAGCAGTTTGCAGTTGGAGAAATTATCACT
GATATGGCTAAAAAGGAGTGGAAAGTGGGACTGCCTATTGGACAAGGTGGATTTG
GTTGTATCTATCTGGCCGACATGAACTCTTCGGAATCTGTGGGCTCAGATGCTCC
CTGTGTAGTCAAAGTAGAACCTTCAGATAATGGGCCGCTGTTTACTGAACTGAAAT
TTTATCAAAGGGCTGCTAAACCTGAACAGATACAAAAATGGATACGGACTCGGAAA
TTGAAATATCTCGGCGTACCAAAATATTGGGGTAGCGGACTTCATGATAAAAATGG
GAAATCGTATCGTTTTATGATAATGGACCGGTTCGGCTCGGACTTACAAAAAATTTA
CGAGGCGAACGCCAAACGGTTTAGCCGCAAGACTGTATTACAGCTGAGCTTGCG
CATTCTgGATATTCTTGAgTATATcCACGAACATGAATATGTTCATGGTGATATTAAGG
CAagcAATTTATTATTGAACTATAAGAACCCGGATCAGGTATATTTGGTGGATTACGG
TCTGGCATACCGCTACTGCCCGGAGGGAGTACACAAAGAGTATAAGGAAGACCC
AAAACGGTGTCATGAcGGAACCATCGAATTTACCTCGATAGACGCACATAACGGT
GTCGCGCCCTCACGTCGTGGAGAcCTGGAAATCCTGGGATATTGCATGATTCAGT

GGCTGACGGGACACCTTCCgTGGGAGGATAATCTGAAAGATCCTAAGTATGTGCG<br>AGACAGTAAGATTAGATACAGGGAAAATATAGCCAGCCTGATGGATAAATGCTTTC<br>CaGAAAAGAACAAACCGGGAGAAATCGCTAAATATATGGAGACTGTCAAACTTTTG<br>GATTACACCGAGAAACCGCTGTATGAAAACCTCCGCGATATtTTACTACAGGGCCT<br>GAAAGCCATTGGCAGTAAAGATGATGGCAAGTTAGAcCTGTCAGTGGTTGAAAAC<br>GGGGGTCTTAAAGCAAAGACAATTACGAAAAAACGAAAGAAAGAGATTGAAGAAT<br>CAAAAGAACCAGGCGTTGAAGATACTGAATGGAGCAATACACAGACAGAAGAGG<br>CTATCCAGACGCGTTCCAGAACCCGCAAACGTGTTCAGAAGAGCGGTCCGAAGA<br>AGAAACGGAAAGTTGGTACCAGTGCACGAAAGCGCGAAGTACGCctgATGAAaAA<br>CCGCGAAGCAGCTAGAGAGTGTCGTCGTAAgAAgAAgGAATATGTTAAGTGCCTT<br>GAGAACCGGGTGGCTGTaCTGGAGAATCAGAACAAgACGTTGATCGAAGAACTTA<br>AGGCGCTTAAGGACCTGTATTGCCACAAATCCGACTAA
**bZIP$_{CREB}$ for N-terminal fusion gene sequence**<br>TATACCATGGGGAGtTCACACCATCATCACCACCAcGGATCTGGTGAgAATTTGTAT<br>TTcCAAAGTGCACGAAAGCGCGAAGTACGCctgATGAAaAACCGCGAAGCAGCTAG<br>AGAGTGTCGTCGTAAgAAgAAgGAATATGTTAAGTGCCTTGAGAACCGGGTGGCT<br>GTaCTGGAGAATCAGAACAAgACGTTGATCGAAGAACTTAAGGCGCTTAAGGACC<br>TGTATTGCCACAAATCCGACGCTAGCacc
**bZIP$_{CREB}$ for C-terminal fusion gene sequence**<br>GGTGGTACCAGTGCACGAAAGCGCGAAGTACGCctgATGAAaAACCGCGAAGCAG<br>CTAGAGAGTGTCGTCGTAAgAAgAAgGAATATGTTAAGTGCCTTGAGAACCGGGTG<br>GCTGTaCTGGAGAATCAGAACAAgACGTTGATCGAAGAACTTAAGGCGCTTAAGG<br>ACCTGTATTGCCACAAATCCGACTAACTCGAGCAC
**bZIP$_{CREB-E319K}$ for C-terminal fusion gene sequence**<br>GGTGGTACCAGTGCACGAAAGCGCGAAGTACGCctgATGAAaAACCGCGAAGCAG<br>CTAGAGAGTGTCGTCGTAAgAAgAAgGAATATGTTAAGTGCCTTGAGAACGATGTG<br>GCTGTaCTGAAGAATCAGAACAAgACGTTGATCGAAGAACTTAAGGCGCTTAAGG<br>ACCTGTATTGCCACAAATCCGACTAACTCGAGCAC
**bZIP$_{BACH1}$ for C-terminal fusion gene sequence**<br>GGTGGTACCAGTCATAAACTAACTCCGGAACAGCTAGACTGTATTCACGACATAC<br>GACGCCGCTCAAAGAATCGGATTGCTGCTCAAAGATGCCGCAAAAGAAAATTAGA<br>CTGCATTCAGAATCTGGAATCTGAAATTGAGAAACTTCAATCGGAGAAAGAATCTT<br>TATTGAAAGAACGGGATCATATCTTAAGCACATTAGGCGAAACCAAACAGAATTTA<br>ACGGGTCTATGCCAAAAAGTGTAACTCGAGCAC
**bZIP$_{ATF3}$ for C-terminal fusion gene sequence**<br>GGTGGTACCAGTACCAAGGCAGAGGTTGCGCCAGAAGAGGATGAACGTAAGAAA<br>AGGCGCCGTGAGCGCAATAAAATAGCAGCCGCCAAATGTCGGAATAAAAAAAAAG<br>AAAAAACCGAATGCCTGCAAAAAGAATCAGAAAAACTTGAGTCCGTAAATGCGGA<br>GTTGAAGGCACAGATTGAAGAGCTGAAGAATGAAAAACAGCACCTTATCTATATGC<br>TTAATCTGCATCGGTAACTCGAGCAC
**bZIP$_{BATF}$ for C-terminal fusion gene sequence**<br>GGTGGTACCAGTCAACCGCAGCAGCAGTCGCCGGAAGATGATGACCGGAAAGTA<br>AGACGCCGTGAAAAAAACCGTGTTGCAGCGCAACGTAGCCGTAAGAAACAGACC<br>CAGAAAGCAGATAAGTTGCATGAGGAATATGAAAGCCTGGAACAGGAAAATACTAT<br>GTTGCGCCGCGAAATTGGTAAACTGACGGAGGAACTTAAACATCTGACAGAAGC<br>ATTGAAAGAACACGAATAACTCGAGCAC

muGFP-bZIP$_{CREB}$ gene sequence

ATGCATCATCACCACCATCACGGATCCAGTAAAGGAGAAGAATTATTTACGGGTGT
TGTTCCGATCCTGGTTGAGCTGGACGGCGACGTTAATGGTCATAAGTTCTCAGTT
CGAGGTGAGGGTGAAGGCGATGCTACCAACGGCAAACTGACGCTGAAGTTTATT
TGTACCACAGGAAAACTCCCAGTGCCTTGGCCAACACTAGTAACAACCCTGACTT
ACGGCGTGCTGTGTTTCAGTCGTTATCCTGATCACATGAAACGTCATGACTTCTTC
AAATCCGCAATGCCCGAGGGATATGTTCAGGAGAGAACAATTAGCTTTAAAGATGA
TGGTACTTATAAAACCCGGGCCGAGGTGAAGTTCGAAGGCGACACATTAGTCAAT
CGGATTGAATTGAAAGGGATAGATTTCAAAGAAGACGGGAATATTCTGGGCCATAA
GTTAGAATATAACTTTAATTCACATAATGTATACATTACTGCTGATAAGCAGAAAAAC
GGTATCAAGGCATATTTCAAGATTCGCCATAACGTCGAAGATGGTAGTGTACAACT
TGCTGATCATTATCAGCAGAATACGCCGATCGGGGATGGACCTGTACTGTTACCG
GATAACCACTATTTATCTACGCAGTCGGTTTTGAGCAAGGACCCAAACGAAAAAC
GCGACCACATGGTACTGCTTGAGGATGTAACCGCGGCGGGTATCACACATGGTAT
GGATGAATTATATAAGGGGGGAGGCAGCGGTGGTACCAGTGCACGAAAGCGCGA
AGTACGCctgATGAAaAACCGCGAAGCAGCTAGAGAGTGTCGTCGTAAgAAgAAgG
AATATGTTAAGTGCCTTGAGAACCGGGTGGCTGTaCTGGAGAATCAGAACAAgAC
GTTGATCGAAGAACTTAAGGCGCTTAAGGACCTGTATTGCCACAAATCCGACTAA

muGFP-bZIP$_{CREB-E319K}$ gene sequence

ATGCATCATCACCACCATCACGGATCCAGTAAAGGAGAAGAATTATTTACGGGTGT
TGTTCCGATCCTGGTTGAGCTGGACGGCGACGTTAATGGTCATAAGTTCTCAGTT
CGAGGTGAGGGTGAAGGCGATGCTACCAACGGCAAACTGACGCTGAAGTTTATT
TGTACCACAGGAAAACTCCCAGTGCCTTGGCCAACACTAGTAACAACCCTGACTT
ACGGCGTGCTGTGTTTCAGTCGTTATCCTGATCACATGAAACGTCATGACTTCTTC
AAATCCGCAATGCCCGAGGGATATGTTCAGGAGAGAACAATTAGCTTTAAAGATGA
TGGTACTTATAAAACCCGGGCCGAGGTGAAGTTCGAAGGCGACACATTAGTCAAT
CGGATTGAATTGAAAGGGATAGATTTCAAAGAAGACGGGAATATTCTGGGCCATAA
GTTAGAATATAACTTTAATTCACATAATGTATACATTACTGCTGATAAGCAGAAAAAC
GGTATCAAGGCATATTTCAAGATTCGCCATAACGTCGAAGATGGTAGTGTACAACT
TGCTGATCATTATCAGCAGAATACGCCGATCGGGGATGGACCTGTACTGTTACCG
GATAACCACTATTTATCTACGCAGTCGGTTTTGAGCAAGGACCCAAACGAAAAAC
GCGACCACATGGTACTGCTTGAGGATGTAACCGCGGCGGGTATCACACATGGTAT
GGATGAATTATATAAGGGGGGAGGCAGCGGTGGTACCAGTGCACGAAAGCGCGA
AGTACGCctgATGAAaAACCGCGAAGCAGCTAGAGAGTGTCGTCGTAAgAAgAAgG
AATATGTTAAGTGCCTTGAGAACCGGGTGGCTGTaCTGAAGAATCAGAACAAgAC
GTTGATCGAAGAACTTAAGGCGCTTAAGGACCTGTATTGCCACAAATCCGACTAA

Full plasmid sequence of ccTFAM-VRK1

AATACGACTCACTATAGGGGAATTGTGAGCGGATAACAATTCCCCTCTAGAAATAA
TTTTGTTTAACTTTAAGAAGGAGATATACCATGGGGAGTTCACACCATCATCACCA
CCACGGATCTGGTAGTATGAGTTCAGTGCTGGCTAGCTGTCCGAAAAAACCAGTC
TCTTCATATCTGCGTTTTTCAAAAGAGCAGTTGCCAATCTTTAAGGCCCAAAATCC
AGATGCGAAAACAACTGAGCTGATTAGACGCATAGCGCAACGGTGGAGAGAACT
GCCGGACTCCAAGAAGAAGATTTATCAGGACGCGTATCGCTGTGAGTGGCAATG
CTATAAAGAAGAAATATCGCGTTTCAAAGAACAGCTGACCCCTAGTCAGATTATGT
CCCTTGAGAAAGAAATCATGGATAAACACCTGAAACGAAAAGCAATGACCAAGAA
AAAAGAATTAACCTTACTGGGAAAACCAAAGCGGCCGCGCAGTGCATACAATGTT

TATGTGGCTGAACGGTTTCAAGAGGCAAAAGGCGATTCTCCTCAGGAGAAACTG
AAAACGGTTAAAGAAAATTGGAAGAACCTCTCCGATTCAGAGAAGGAACTGTATAT
CCAGCACGCTAAAGAGGATGAAACAAGATATCATAACGAAATGAAATCCTGGGAG
GAGCAGATGATTGAGGTAGGTCGGAAAGACCTTCTACGTCGCACTATTAAAAAAC
AGCGCAAATACGGTGCTGAAGAATGCAGTGGGGGTAGCTCCGGCCGTGGATCCA
TGCCCCGTGTGAAGGCGGCGCAGGCTGGACGGCAGTCTTCAGCGAAGCGTCAC
CTCGCGGAGCAGTTTGCAGTTGGAGAAATTATCACTGATATGGCTAAAAAGGAGT
GGAAAGTGGGACTGCCTATTGGACAAGGTGGATTTGGTTGTATCTATCTGGCCGA
CATGAACTCTTCGGAATCTGTGGGCTCAGATGCTCCCTGTGTAGTCAAAGTAGAA
CCTTCAGATAATGGGCCGCTGTTTACTGAACTGAAATTTTATCAAAGGGCTGCTAA
ACCTGAACAGATACAAAAATGGATACGGACTCGGAAATTGAAATATCTCGGCGTAC
CAAAATATTGGGGTAGCGGACTTCATGATAAAAATGGGAAATCGTATCGTTTTATGA
TAATGGACCGGTTCGGCTCGGACTTACAAAAAATTTACGAGGCGAACGCCAAACG
GTTTAGCCGCAAGACTGTATTACAGCTGAGCTTGCGCATTCTGGATATTCTTGAGT
ATATCCACGAACATGAATATGTTCATGGTGATATTAAGGCAAGCAATTTATTATTGAA
CTATAAGAACCCGGATCAGGTATATTTGGTGGATTACGGTCTGGCATACCGCTACT
GCCCGGAGGGAGTACACAAAGAGTATAAGGAAGACCCAAAACGGTGTCATGACG
GAACCATCGAATTTACCTCGATAGACGCACATAACGGTGTCGCGCCCTCACGTCG
TGGAGACCTGGAAATCCTGGGATATTGCATGATTCAGTGGCTGACGGGACACCTT
CCGTGGGAGGATAATCTGAAAGATCCTAAGTATGTGCGAGACAGTAAGATTAGATA
CAGGGAAAATATAGCCAGCCTGATGGATAAATGCTTTCCAGAAAAGAACAAACCG
GGAGAAATCGCTAAATATATGGAGACTGTCAAACTTTTGGATTACACCGAGAAACC
GCTGTATGAAAACCTCCGCGATATTTTACTACAGGGCCTGAAAGCCATTGGCAGTA
AAGATGATGGCAAGTTAGACCTGTCAGTGGTTGAAAACGGGGGTCTTAAAGCAAA
GACAATTACGAAAAAACGAAAGAAAGAGATTGAAGAATCAAAAGAACCAGGCGTT
GAAGATACTGAATGGAGCAATACACAGACAGAAGAGGCTATCCAGACGCGTTCCA
GAACCCGCAAACGTGTTCAGAAGAGCGGTCCGAAGAAGAAACGGAAAGTATAAC
TCGAGCACCACCACCACCACCACTGAGATCCGGCTGCTAACAAAGCCCGAAAGG
AAGCTGAGTTGGCTGCTGCCACCGCTGAGCAATAACTAGCATAACCCCTTGGGG
CCTCTAAACGGGTCTTGAGGGGTTTTTTGCTGAAAGGAGGAACTATATCCGGATT
GGCGAATGGGACGCGCCCTGTAGCGGCGCATTAAGCGCGGCGGGTGTGGTGGT
TACGCGCAGCGTGACCGCTACACTTGCCAGCGCCCTAGCGCCCGCTCCTTTCGC
TTTCTTCCCTTCCTTTCTCGCCACGTTCGCCGGCTTTCCCCGTCAAGCTCTAAAT
CGGGGGCTCCCTTTAGGGTTCCGATTTAGTGCTTTACGGCACCTCGACCCCAAA
AAACTTGATTAGGGTGATGGTTCACGTAGTGGGCCATCGCCCTGATAGACGGTTT
TTCGCCCTTTGACGTTGGAGTCCACGTTCTTTAATAGTGGACTCTTGTTCCAAACT
GGAACAACACTCAACCCTATCTCGGTCTATTCTTTTGATTTATAAGGGATTTTGCCG
ATTTCGGCCTATTGGTTAAAAAATGAGCTGATTTAACAAAAATTTAACGCGAATTTT
AACAAACTAGTAACGTTTACAATTTCAGGTGGCACTTTTCGGGGAAATGTGCGCG
GAACCCCTATTTGTTTATTTTTCTAAATACATTCAAATATGTATCCGCTCATGAATTAA
TTCTTAGAAAAACTCATCGAGCATCAAATGAAACTGCAATTTATTCATATCAGGATT
ATCAATACCATATTTTTGAAAAAGCCGTTTCTGTAATGAAGGAGAAAACTCACCGA
GGCAGTTCCATAGGATGGCAAGATCCTGGTATCGGTCTGCGATTCCGACTCGTCC
AACATCAATACAACCTATTAATTTCCCCTCGTCAAAAATAAGGTTATCAAGTGAGAA
ATCACCATGAGTGACGACTGAATCCGGTGAGAATGGCAAAAGTTTATGCATTTCTT
TCCAGACTTGTTCAACAGGCCAGCCATTACGCTCGTCATCAAAATCACTCGCATC

AACCAAACCGTTATTCATTCGTGATTGCGCCTGAGCGAGACGAAATACGCGATCG
CTGTTAAAAGGACAATTACAAACAGGAATCGAATGCAACCGGCGCAGGAACACTG
CCAGCGCATCAACAATGTTTTCACCTGAATCAGGATATTCTTCTAATACCTGGAAT
GCTGTTTTCCCGGGGATCGCAGTGGTGAGTAACCATGCATCATCAGGAGTACGG
ATAAAATGCTTGATGGTCGGAAGAGGCATAAATTCCGTCAGCCAGTTTAGTCTGAC
CATCTCATCTGTAACATCATTGGCAACGCTACCTTTGCCATGTTTCAGAAACAACT
CTGGCGCATCGGGCTTCCCATACAATCGATAGATTGTCGCACCTGATTGCCCGAC
ATTATCGCGAGCCCATTTATACCCATATAAATCAGCATCCATGTTGGAATTTAATCG
CGGCCTAGAGCAAGACGTTTCCCGTTGAATATGGCTCATAACACCCCTTGTATTAC
TGTTTATGTAAGCAGACAGTTTTATTGTTCATGACCAAAATCCCTTAACGTGAGTTT
TCGTTCCACTGAGCGTCAGACCCCGTAGAAAAGATCAAAGGATCTTCTTGAGATC
CTTTTTTTCTGCGCGTAATCTGCTGCTTGCAAACAAAAAAACCACCGCTACCAGC
GGTGGTTTGTTTGCCGGATCAAGAGCTACCAACTCTTTTTCCGAAGGTAACTGGC
TTCAGCAGAGCGCAGATACCAAATACTGTCCTTCTAGTGTAGCCGTAGTTAGGCC
ACCACTTCAAGAACTCTGTAGCACCGCCTACATACCTCGCTCTGCTAATCCTGTTA
CCAGTGGCTGCTGCCAGTGGCGATAAGTCGTGTCTTACCGGGTTGGACTCAAGA
CGATAGTTACCGGATAAGGCGCAGCGGTCGGGCTGAACGGGGGGTTCGTGCAC
ACAGCCCAGCTTGGAGCGAACGACCTACACCGAACTGAGATACCTACAGCGTGA
GCTATGAGAAAGCGCCACGCTTCCCGAAGGGAGAAAGGCGGACAGGTATCCGG
TAAGCGGCAGGGTCGGAACAGGAGAGCGCACGAGGGAGCTTCCAGGGGGAAA
CGCCTGGTATCTTTATAGTCCTGTCGGGTTTCGCCACCTCTGACTTGAGCGTCGA
TTTTTGTGATGCTCGTCAGGGGGGCGGAGCCTATGGAAAAACGCCAGCAACGCG
GCCTTTTTACGGTTCCTGGCCTTTTGCTGGCCTTTTGCTCACATGTTCTTTCCTGC
GTTATCCCCTGATTCTGTGGATAACCGTATTACCGCCTTTGAGTGAGCTGATACCG
CTCGCCGCAGCCGAACGACCGAGCGCAGCGAGTCAGTGAGCGAGGAAGCGGA
AGAGCGCCTGATGCGGTATTTTCTCCTTACGCATCTGTGCGGTATTTCACACCGC
ATATATGGTGCACTCTCAGTACAATCTGCTCTGATGCCGCATAGTTAAGCCAGTATA
CACTCCGCTATCGCTACGTGACTGGGTCATGGCTGCGCCCCGACACCCGCCAAC
ACCCGCTGACGCGCCCTGACGGGCTTGTCTGCTCCCGGCATCCGCTTACAGAC
AAGCTGTGACCGTCTCCGGGAGCTGCATGTGTCAGAGGTTTTCACCGTCATCAC
CGAAACGCGCGAGGCAGCTGCGGTAAAGCTCATCAGCGTGGTCGTGAAGCGAT
TCACAGATGTCTGCCTGTTCATCCGCGTCCAGCTCGTTGAGTTTCTCCAGAAGCG
TTAATGTCTGGCTTCTGATAAAGCGGGCCATGTTAAGGGCGGTTTTTTCCTGTTTG
GTCACTGATGCCTCCGTGTAAGGGGGATTTCTGTTCATGGGGGTAATGATACCGA
TGAAACGAGAGAGGATGCTCACGATACGGGTTACTGATGATGAACATGCCCGGTT
ACTGGAACGTTGTGAGGGTAAACAACTGGCGGTATGGATGCGGCGGGACCAGA
GAAAAATCACTCAGGGTCAATGCCAGCGCTTCGTTAATACAGATGTAGGTGTTCC
ACAGGGTAGCCAGCAGCATCCTGCGATGCAGATCCGGAACATAATGGTGCAGGG
CGCTGACTTCCGCGTTTCCAGACTTTACGAAACACGGAAACCGAAGACCATTCAT
GTTGTTGCTCAGGTCGCAGACGTTTTGCAGCAGCAGTCGCTTCACGTTCGCTCG
CGTATCGGTGATTCATTCTGCTAACCAGTAAGGCAACCCCGCCAGCCTAGCCGG
GTCCTCAACGACAGGAGCACGATCATGCGCACCCGTGGGGCCGCCATGCCGGC
GATAATGGCCTGCTTCTCGCCGAAACGTTTGGTGGCGGGACCAGTGACGAAGGC
TTGAGCGAGGGCGTGCAAGATTCCGAATACCGCAAGCGACAGGCCGATCATCGT
CGCGCTCCAGCGAAAGCGGTCCTCGCCGAAAATGACCCAGAGCGCTGCCGGCA
CCTGTCCTACGAGTTGCATGATAAAGAAGACAGTCATAAGTGCGGCGACGATAGT

CATGCCCCGCGCCCACCGGAAGGAGCTGACTGGGTTGAAGGCTCTCAAGGGCA
TCGGTCGAGATCCCGGTGCCTAATGAGTGAGCTAACTTACATTAATTGCGTTGCG
CTCACTGCCCGCTTTCCAGTCGGGAAACCTGTCGTGCCAGCTGCATTAATGAATC
GGCCAACGCGCGGGGAGAGGCGGTTTGCGTATTGGGCGCCAGGGTGGTTTTTC
TTTTCACCAGTGAGACGGGCAACAGCTGATTGCCCTTCACCGCCTGGCCCTGAG
AGAGTTGCAGCAAGCGGTCCACGCTGGTTTGCCCCAGCAGGCGAAAATCCTGTT
TGATGGTGGTTAACGGCGGGATATAACATGAGCTGTCTTCGGTATCGTCGTATCC
CACTACCGAGATATCCGCACCAACGCGCAGCCCGGACTCGGTAATGGCGCGCAT
TGCGCCCAGCGCCATCTGATCGTTGGCAACCAGCATCGCAGTGGGAACGATGCC
CTCATTCAGCATTTGCATGGTTTGTTGAAAACCGGACATGGCACTCCAGTCGCCT
TCCCGTTCCGCTATCGGCTGAATTTGATTGCGAGTGAGATATTTATGCCAGCCAGC
CAGACGCAGACGCGCCGAGACAGAACTTAATGGGCCCGCTAACAGCGCGATTTG
CTGGTGACCCAATGCGACCAGATGCTCCACGCCCAGTCGCGTACCGTCTTCATG
GGAGAAAATAATACTGTTGATGGGTGTCTGGTCAGAGACATCAAGAAATAACGCC
GGAACATTAGTGCAGGCAGCTTCCACAGCAATGGCATCCTGGTCATCCAGCGGA
TAGTTAATGATCAGCCCACTGACGCGTTGCGCGAGAAGATTGTGCACCGCCGCT
TTACAGGCTTCGACGCCGCTTCGTTCTACCATCGACACCACCACGCTGGCACCC
AGTTGATCGGCGCGAGATTTAATCGCCGCGACAATTTGCGACGGCGCGTGCAGG
GCCAGACTGGAGGTGGCAACGCCAATCAGCAACGACTGTTTGCCCGCCAGTTGT
TGTGCCACGCGGTTGGGAATGTAATTCAGCTCCGCCATCGCCGCTTCCACTTTTT
CCCGCGTTTTCGCAGAAACGTGGCTGGCCTGGTTCACCACGCGGGAAACGGTC
TGATAAGAGACACCGGCATACTCTGCGACATCGTATAACGTTACTGGTTTCACATT
CACCACCCTGAATTGACTCTCTTCCGGGCGCTATCATGCCATACCGCGAAAGGTT
TTGCGCCATTCGATGGTGTCCGGGATCTCGACGCTCTCCCTTATGCGACTCCTGC
ATTAGGAAGCAGCCCAGTAGTAGGTTGAGGCCGTTGAGCACCGCCGCCGCAAG
GAATGGTGCATGCAAGGAGATGGCGCCCAACAGTCCCCCGGCCACGGGGCCTG
CCACCATACCCACGCCGAAACAAGCGCTCATGAGCCCGAAGTGGCGAGCCCGAT
CTTCCCCATCGGTGATGTCGGCGATATAGGCGCCAGCAACCGCACCTGTGGCGC
CGGTGATGCCGGCCACGATGCGTCCGGCGTAGAGGATCGAGATCTCGATCCCG
CGAAATT

Table S3 – Uniprot accession numbers of all wild-type proteins used in this study

**Protein**	**Accession number**
TFAM	Q00059
VRK1	Q99986
CREB	P16220
BACH1	O14867
ATF3	P18847
BATF	Q16520
GFP	P42212

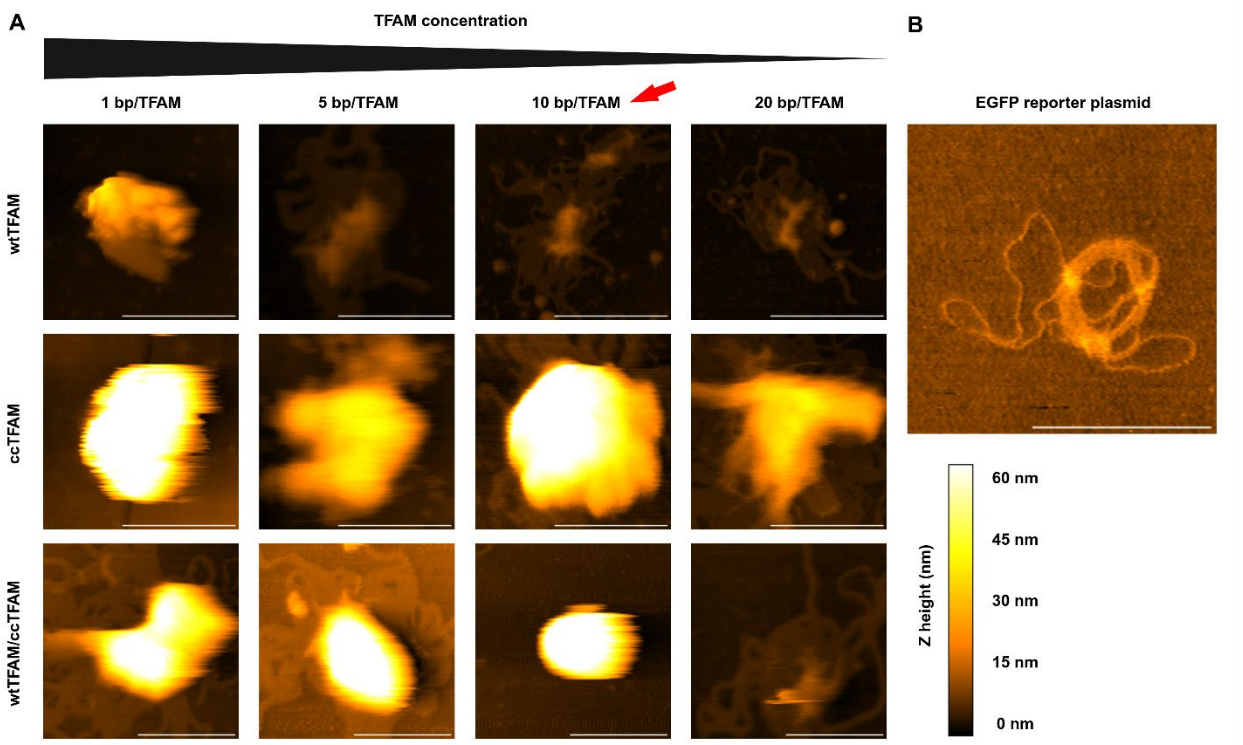

Figure S1 – (A) High-speed atomic force microscopy (HS-AFM) images of TFAM together with immobilized plasmid DNA (pDNA). From left to right: basepairs (bp) per TFAM molecule (bp/TFAM) increases. The upper row shows wildtype TFAM (wtTFAM) with pDNA. The middle row shows ccTFAM with pDNA. The lower row shows an equimolar ratio of wtTFAM and ccTFAM with pDNA. The red arrow indicates the total TFAM concentration in TFAMoplexes. (B) Plasmid DNA in the absence of TFAM. Scale bars, 100 nm.[1]

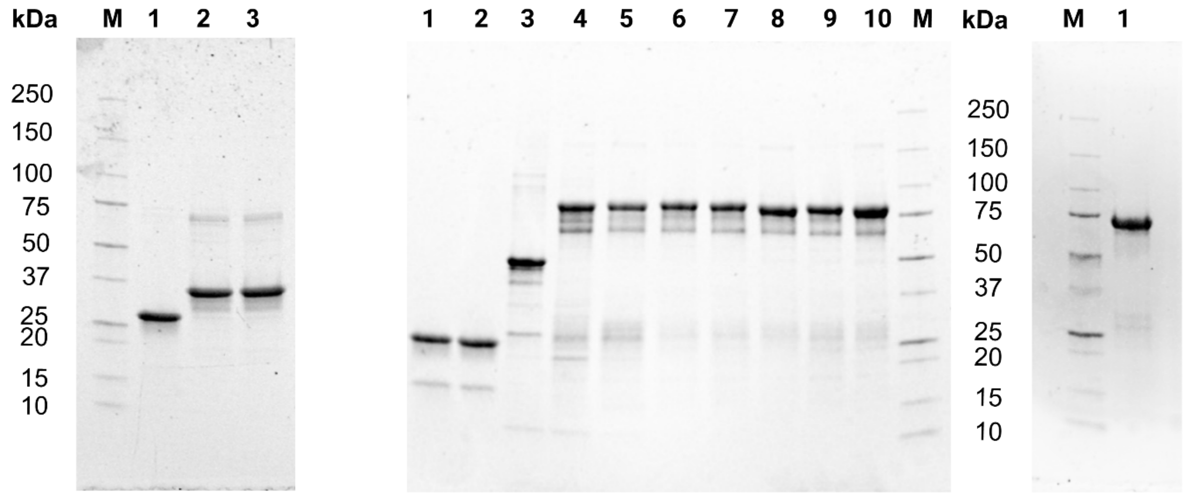

1 muGFP (28 kDa)
2 muGFP-CREB (35 kDa)
3 muGFP-CREB E319K (35 kDa)

1 wtTFAM (28 kDa)
2 ccTFAM (28 kDa)
3 PLC-TFAM (53 kDa)
4 CREB-ccTFAM-VRK1 (81 kDa)
5 ccTFAM-VRK1-CREB (81 kDa)
6 ccTFAM-VRK1-CREB E319K (81 kDa)
7 ccTFAM-VRK1-BACH1 (83 kDa)
8 ccTFAM-VRK1-ATF3 (82 kDa)
9 ccTFAM-VRK1-BATF (82 kDa)

1 ccTFAM-VRK1 (73 kDa)

Figure S2 – SDS-PAGE followed by Coomassie staining of all proteins used in this study. The gel image on the left shows the muGFP-CREB fusions. The gel image on the right shows the TFAM variants. The expected mass of the proteins is indicated in brackets. For all proteins, 4 µg were used.[1]

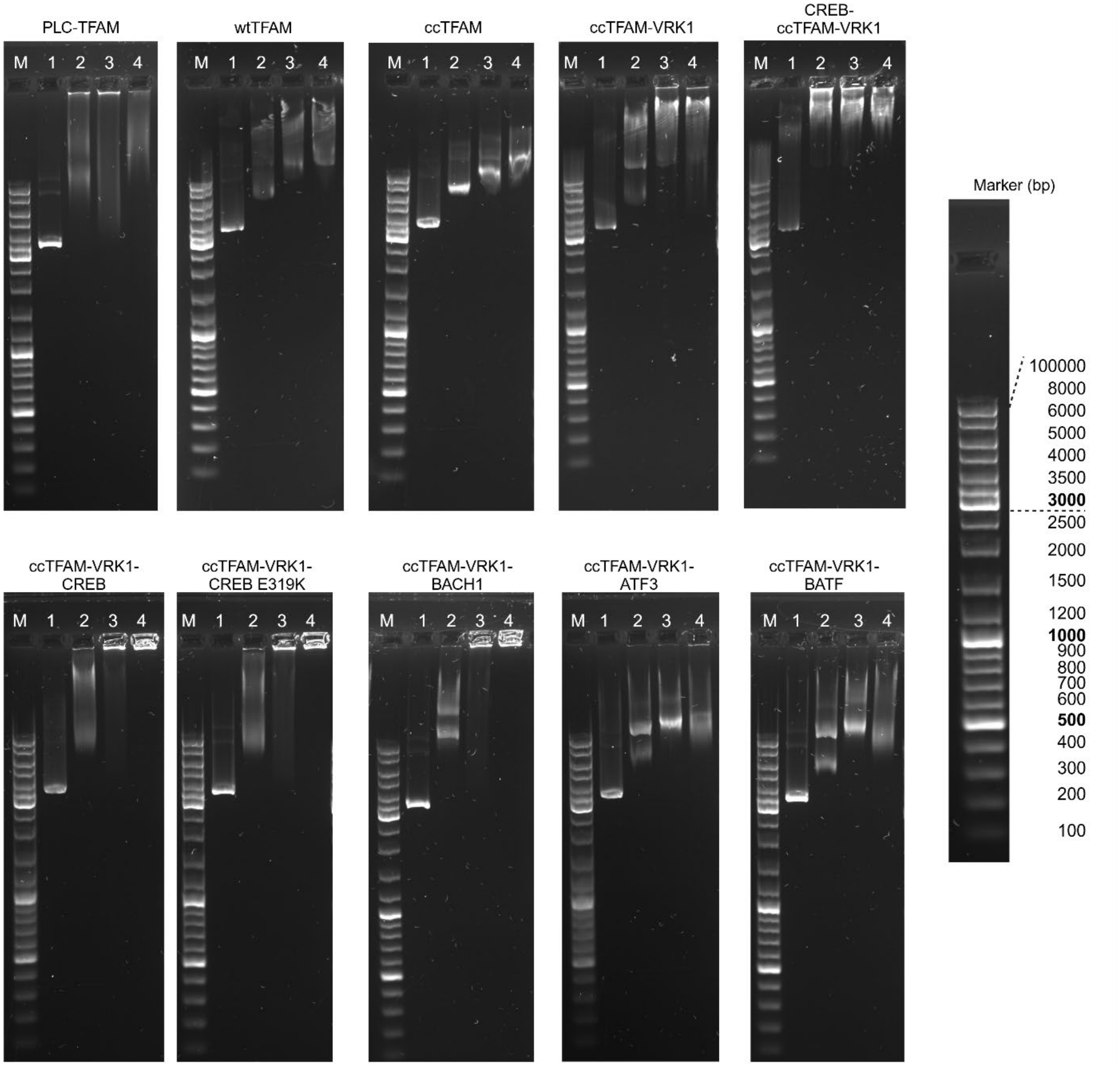

Figure S3 – Gel mobility shift assays of different TFAM-fusion proteins. In each well 100 ng DNA (10 ng/µL) is incubated with 0, 0.25, 0.5, and 1 µM TFAM protein and loaded on lanes 1-4, respectively.[1]

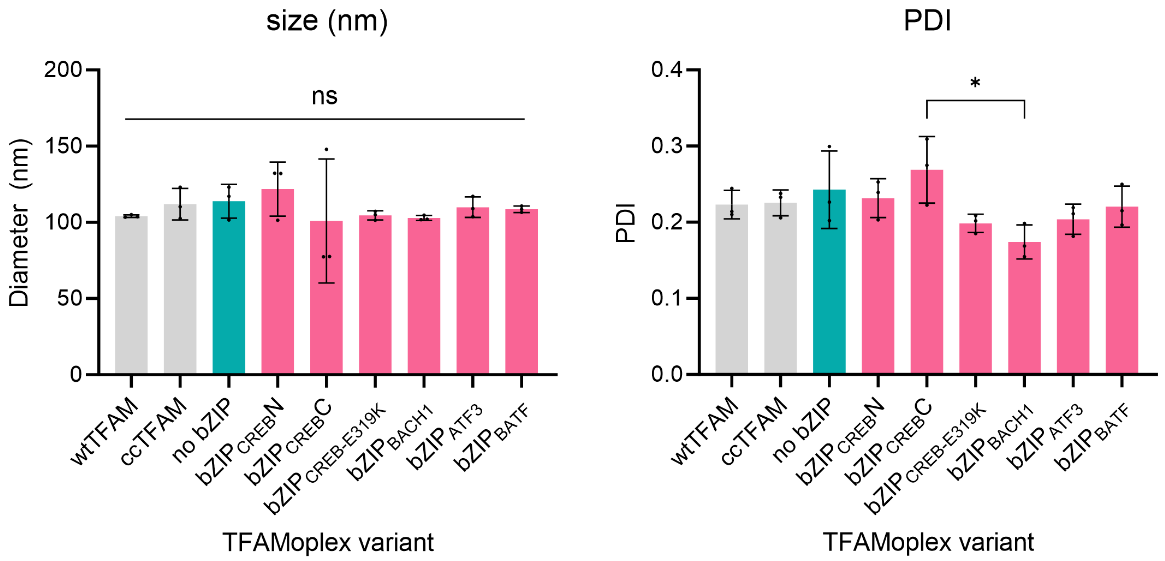

Figure S4.1 – Diameter (left panel) and polydispersity index (PDI, right panel) of different TFAMoplex variants. The indicated proteins were mixed with PLC-TFAM at an equimolar ratio and measured by DLS. The DNA concentration was 10 ng/µL. The bZIP indicates the fusion of the corresponding bZIP domain to ccTFAM-VRK1. The total TFAM concentration is 0.8 µM for all samples. Each dot represents the mean of an independent triplicate experiment. Mean ± SD (N = 3), *$p < 0.05$, **$p < 0.01$, ***$p < 0.001$.

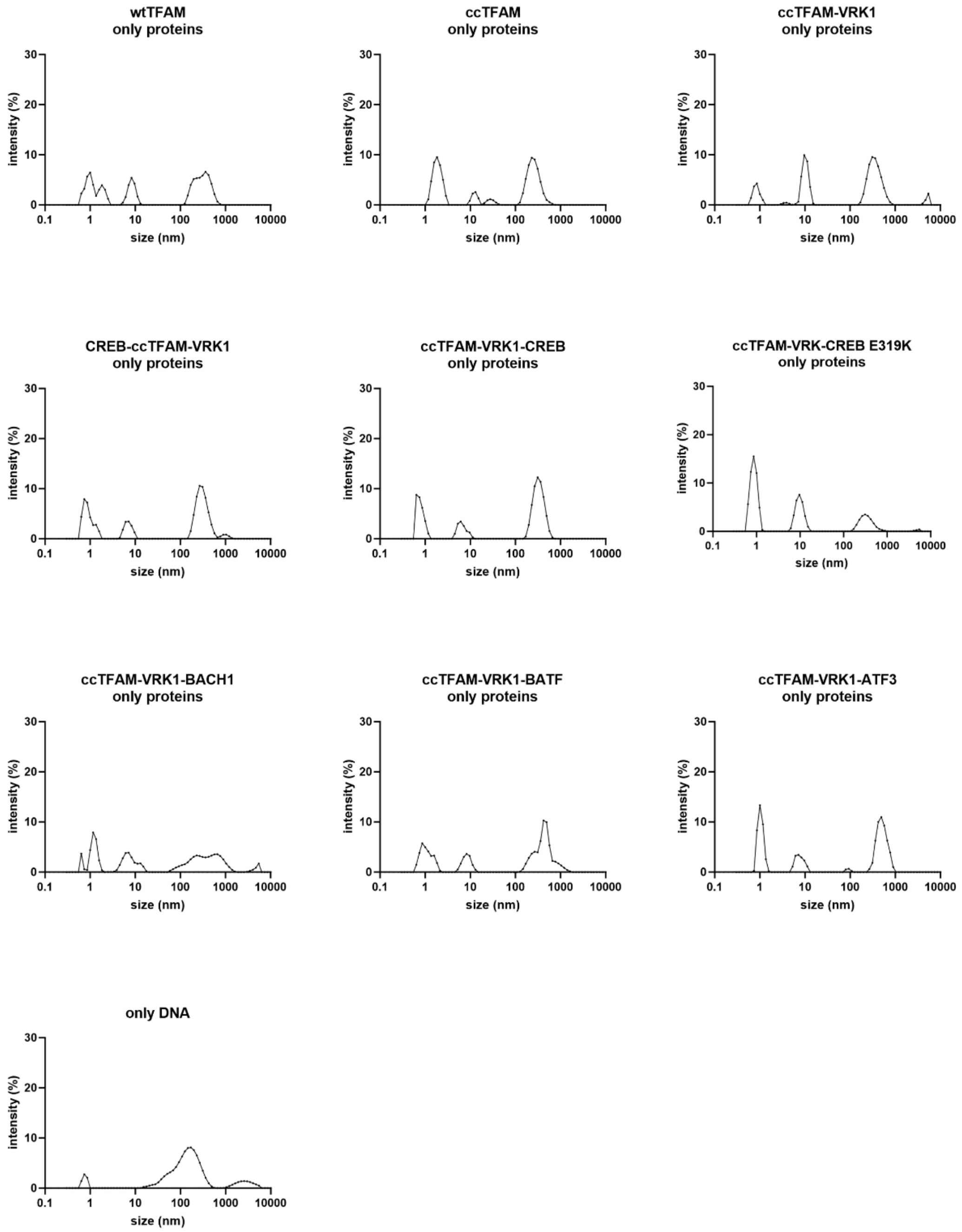

Figure S4.2 - Intensity diagrams of PLC-TFAM and indicated proteins without DNA and DNA without proteins obtained by DLS. The black line represents the mean of 3 independent measurements. Size means the measured hydrodynamic diameter. The DNA concentration was 10 ng/µL in the DNA only sample. The indicated proteins were mixed with PLC-TFAM at an equimolar ratio. The total TFAM concentration is 0.8 µM for all proteins.[1]

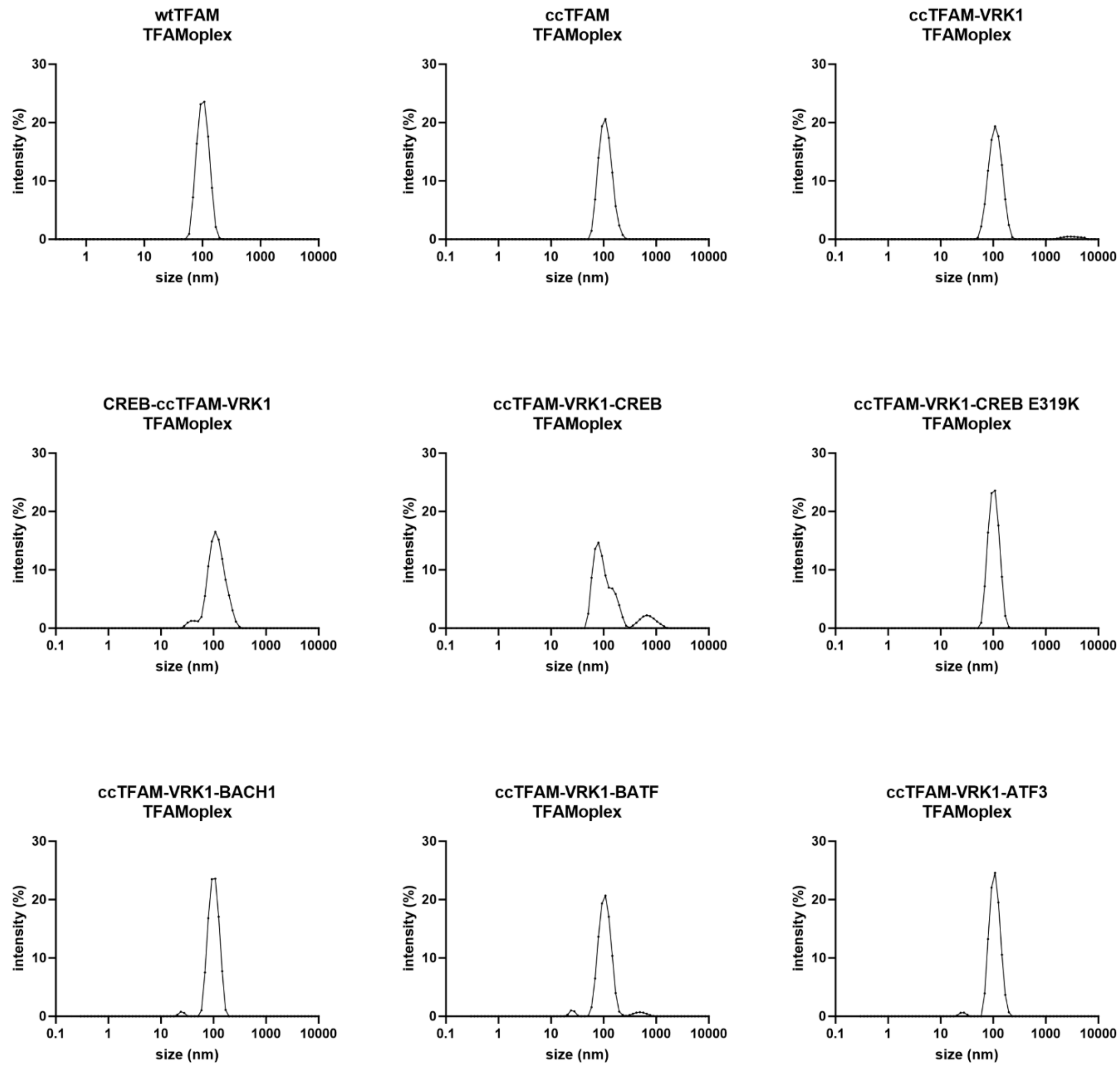

Figure S4.3 - Intensity diagrams of different TFAMoplexes (PLC-TFAM and indicated proteins with DNA) obtained by DLS. The black line represents the mean of 3 independent measurements. Size means the measured hydrodynamic diameter. The DNA concentration was 10 ng/µL in all samples. The indicated proteins were mixed with PLC-TFAM at an equimolar ratio. The total TFAM concentration is 0.8 µM for all proteins.[1]

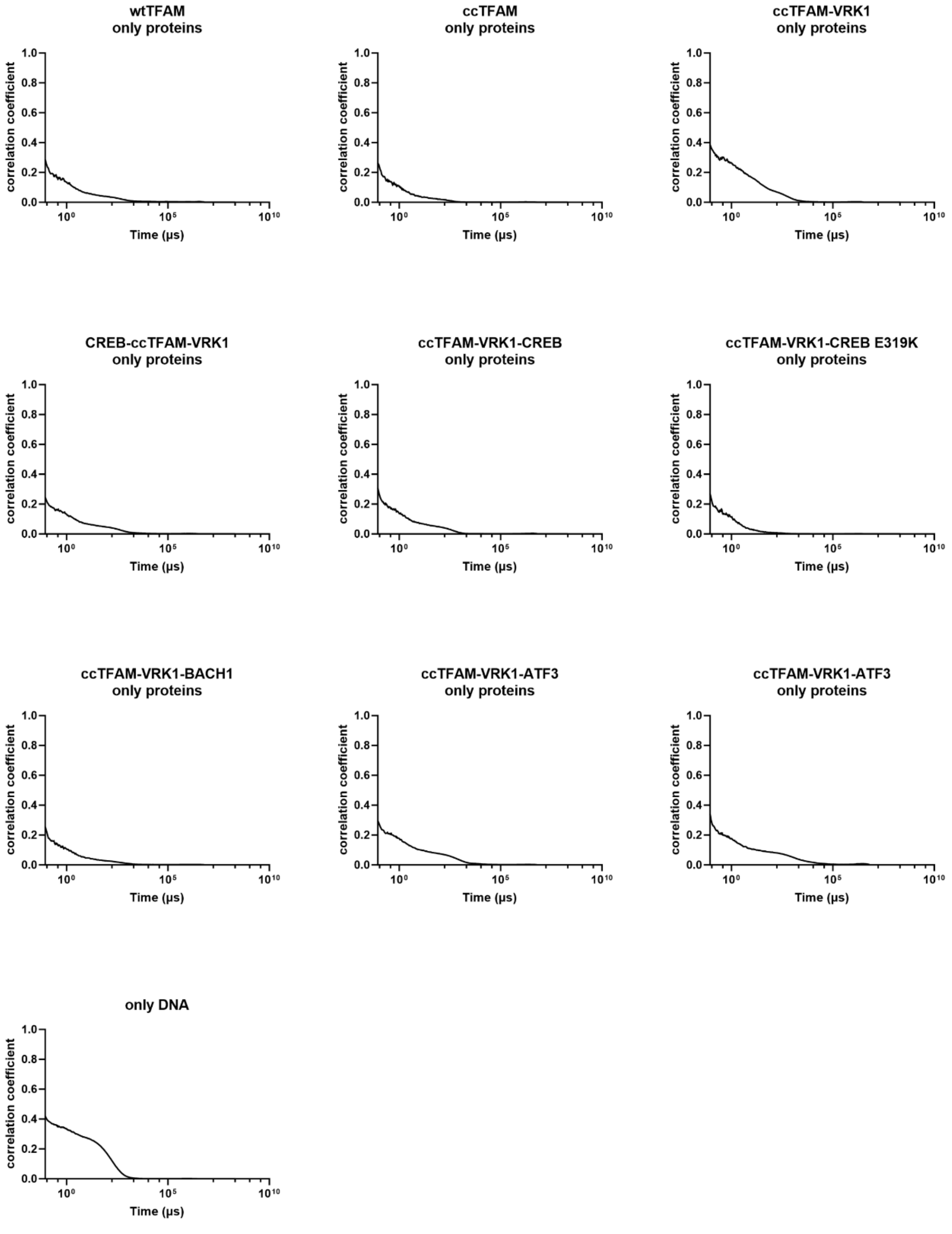

Figure S4.4 - Correlation curves of PLC-TFAM and indicated proteins without DNA and DNA without proteins. The black line represents the mean of 3 independent measurements. The DNA concentration was 10 ng/µL in the DNA only sample. The indicated proteins were mixed with PLC-TFAM at an equimolar ratio. The total TFAM concentration is 0.8 µM for all proteins.[1]

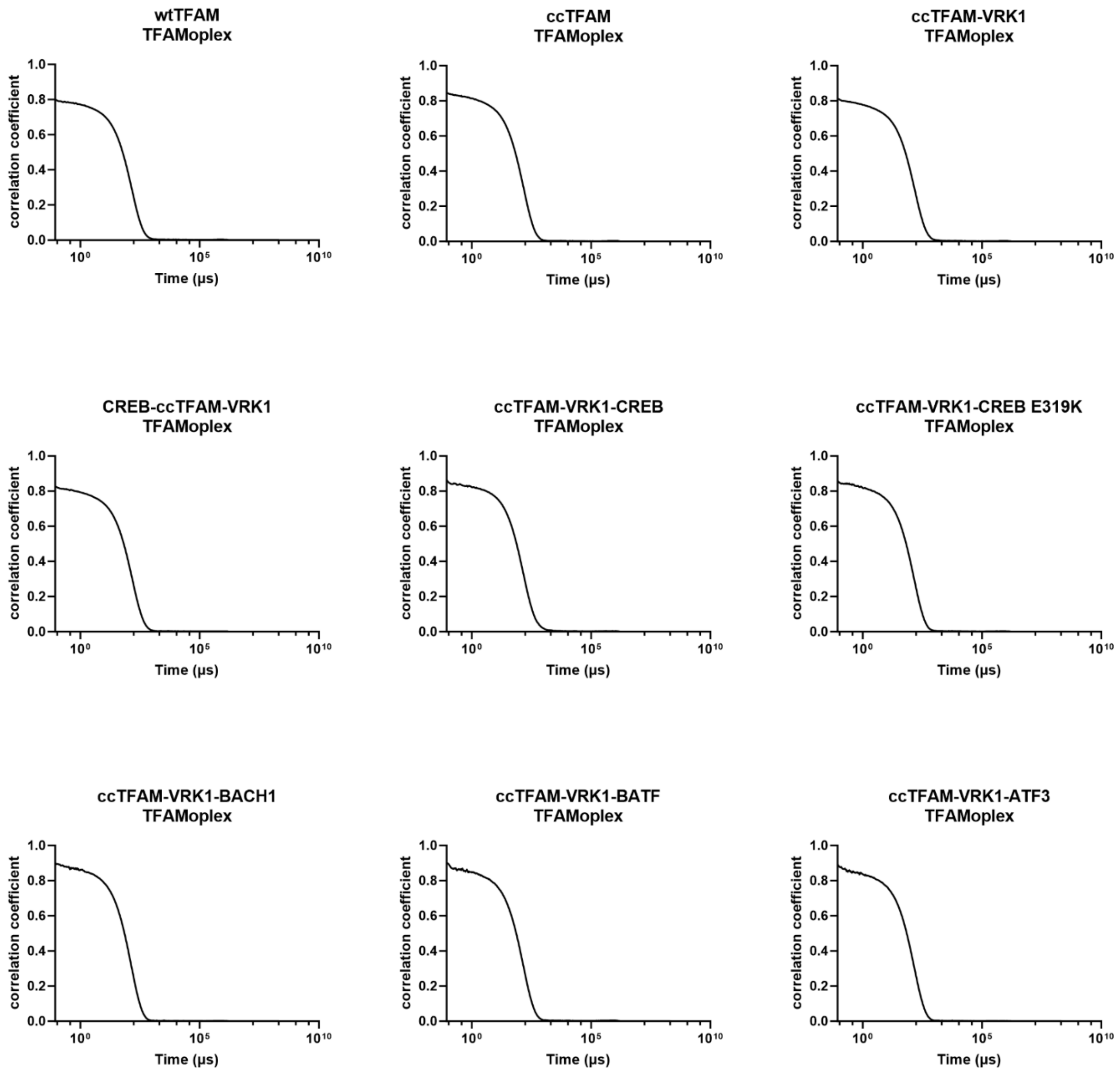

Figure S4.5 - Correlation curves of different TFAMoplexes (PLC-TFAM and indicated proteins with DNA). The black line represents the mean of 3 independent measurements. The DNA concentration was 10 ng/µL in all samples. The indicated proteins were mixed with PLC-TFAM at an equimolar ratio. The total TFAM concentration is 0.8 µM for all proteins.[1]

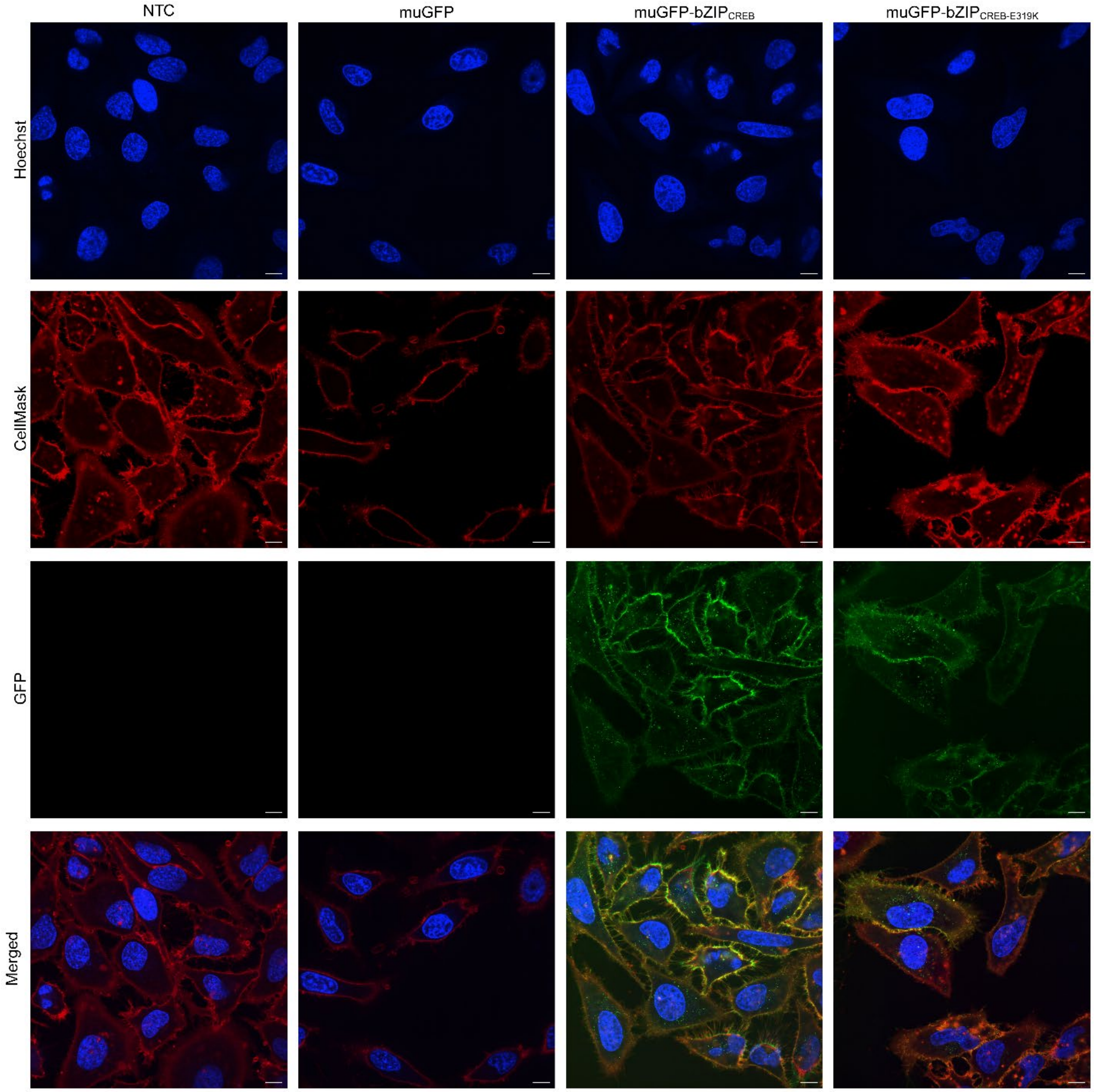

Figure S5 – Uncropped image corresponding to Figure 3. CREB interaction with cell membranes. HeLa cells were incubated with 500 nM of the indicated protein in 100% FBS for 30 min. Column 1: Negative control (NTC) with untreated cells. Column 2: Treatment with 500 nM monomeric ultrastable GFP (muGFP). Column 3: Treatment with muGFP-bZIP$_{CREB}$. Column 4: Treatment with muGFP-bZIP$_{CREB\text{-}E319K}$. Images are shown as single z-slices in different channels. Blue: Hoechst DNA staining. Red: CellMask Deep Red. Green: muGFP signal. Merged: Composite of all channels. Scale bars: 10 µm.[1]

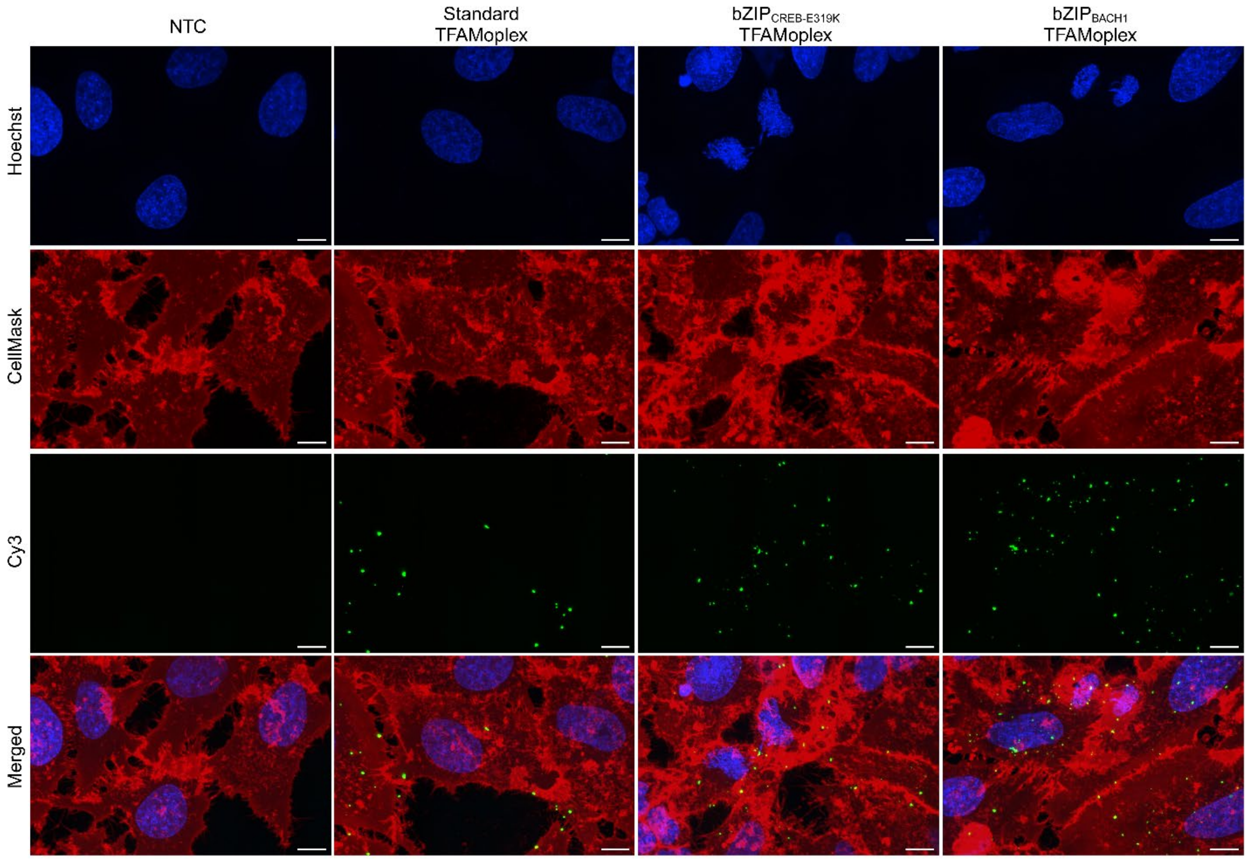

Figure S6 – Corresponding Z-projection image to Figure 4. TFAMoplex association with HeLa cells in 100% FBS 30 min after addition. Various TFAMoplex versions were formed with Cy3-labeled DNA and incubated with cells for 30 min, followed by confocal imaging. Column 1: NTC with untreated cells. Column 2: Standard TFAMoplex. Column 3: $bZIP_{CREB\text{-}E319K}$ TFAMoplex. Column 4: $bZIP_{BACH1}$ TFAMoplex. Blue: Hoechst DNA staining. Red: CellMask Deep Red. Green: Pseudocolored Cy3 signal of the labeled DNA. Merged: Composite of all channels. Scale bars: 10 µm.[1]

Standard plasmid

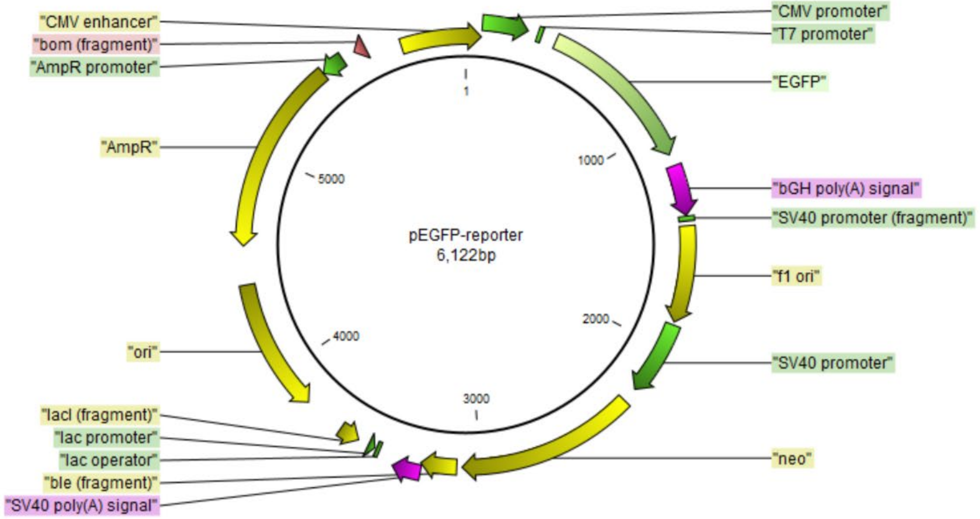

AAV plasmid

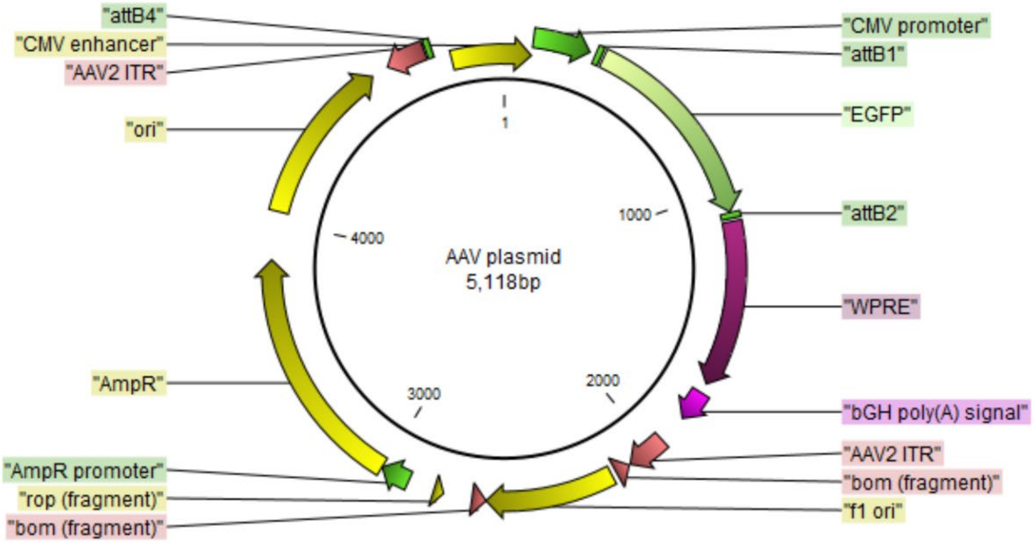

Figure S7 – Plasmid maps of the standard and AAV plasmids. Both plasmids include bacterial elements such as an antibiotic resistance gene and origin of replication, as well as mammalian expression cassettes with a CMV promoter/enhancer, EGFP gene, and poly(A) signal. The AAV plasmid additionally features a WPRE element.[1]

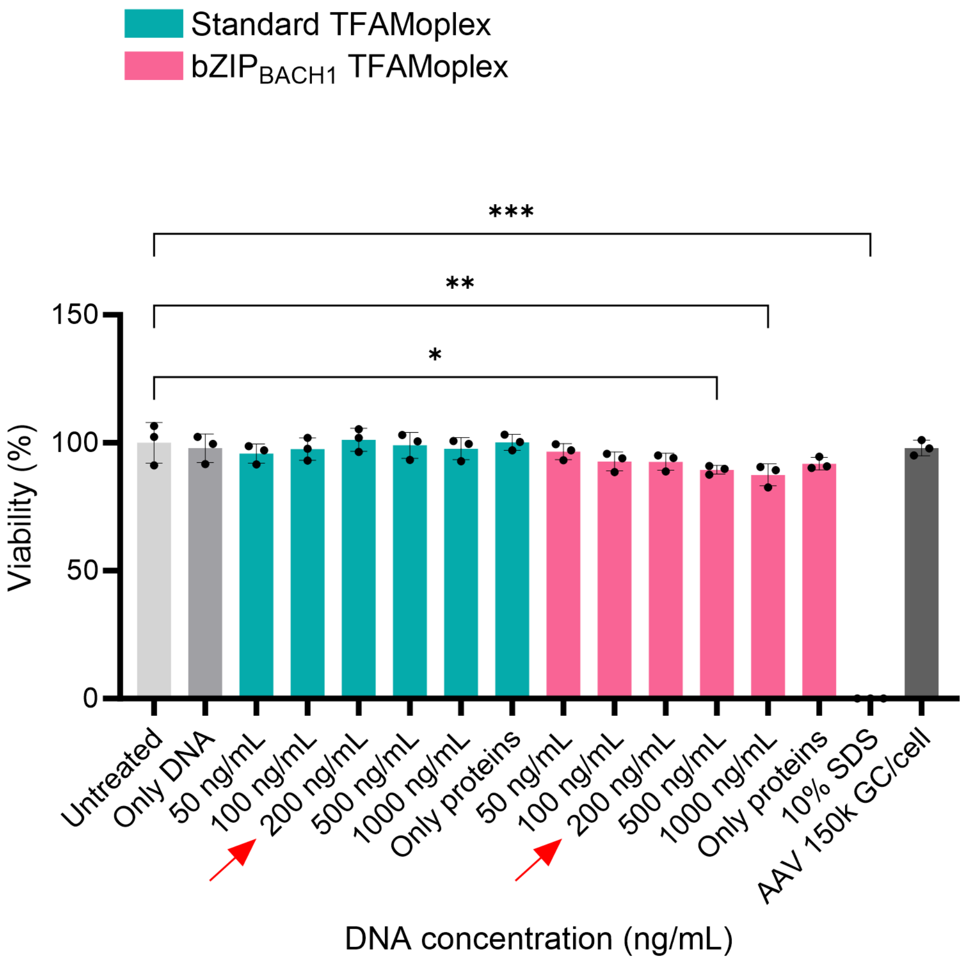

Figure S8 - Cell viability assay comparing two TFAMoplex versions (with and without BACH1 addition) that were used in the determination of EC_{50} values. Both groups contained PLC-TFAM and ccTFAM-fusion proteins (VRK1 and BACH1) at equimolar concentrations. The "only DNA" and "only proteins" control groups were tested at the same concentrations as the "1000 ng/mL" TFAMoplex group. The red arrows indicate the DNA concentration used for the standard transfection experiments described in this study. Sodium dodecyl sulfate (SDS) was used as a positive toxic control. The dark grey bar on the right represents the cell treatment with the highest used AAV concentration in this study. Each dot represents the mean of an independent triplicate experiment. Mean ± SD ($N = 3$), *$p < 0.05$, **$p < 0.01$, ***$p < 0.001$.

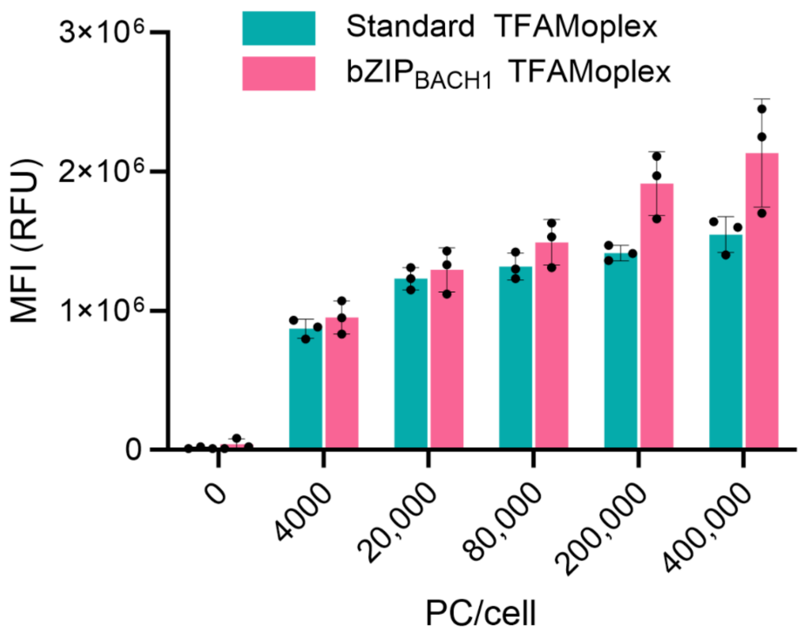

Figure S9 – Corresponding mean fluorescence intensity (MFI) data to the transfection efficiency (TE) data shown in Figure 6. Panel shows the MFI data relating to the transfection efficiency in panel 5B. Each dot represents the mean of an independent triplicate experiment. Mean ± SD (N = 3), *p < 0.05, **p < 0.01, ***p < 0.001.[1]

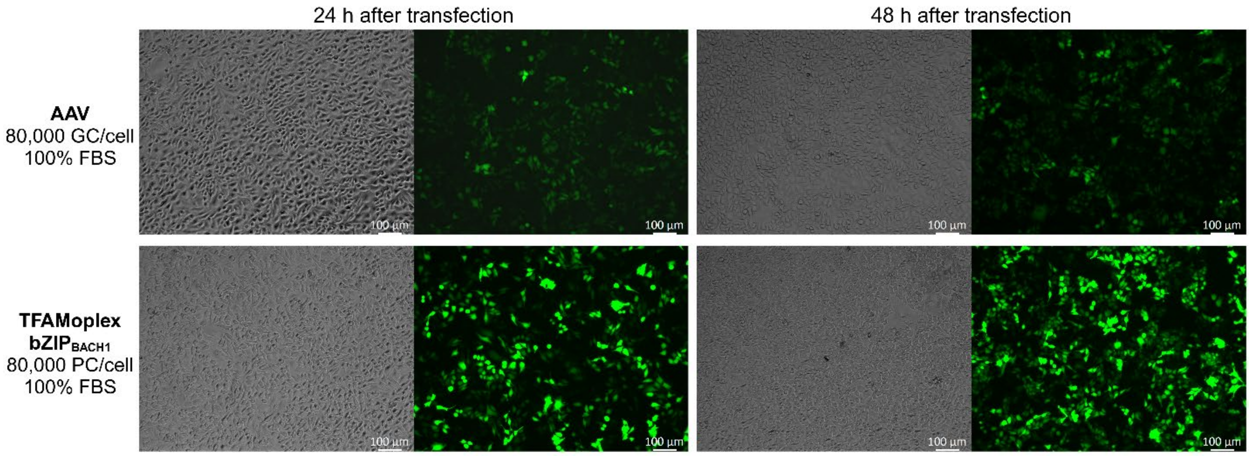

Figure S10 – Microscopy images of HeLa cells transfected with AAV and $bZIP_{BACH1}$-TFAMoplexes. Images were taken 24h and 48h after transfection. The cells were imaged with bright field (left side) and fluorescence microscopy (right side). Scale bar: 100 µm.

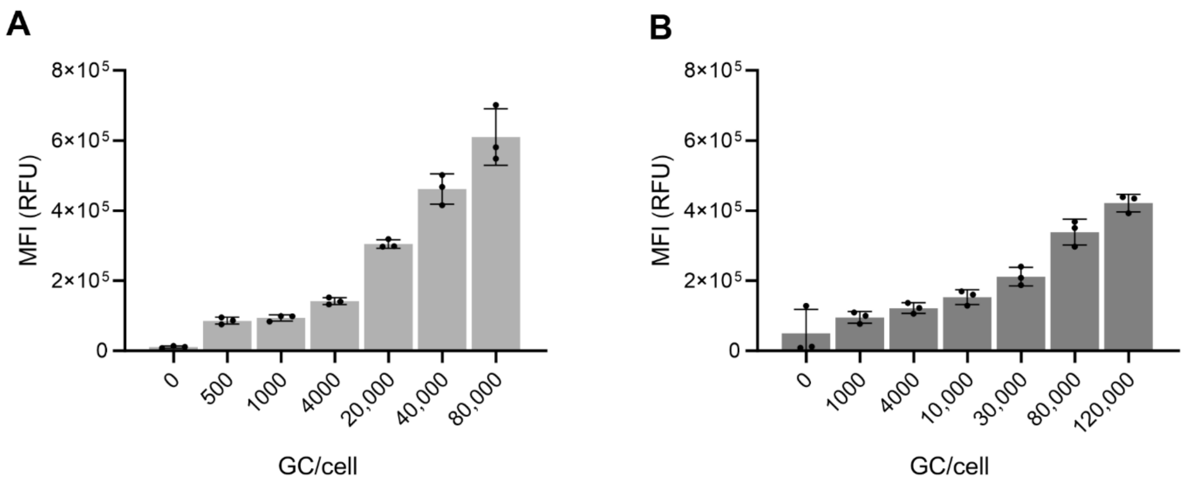

Figure S11 – Corresponding mean fluorescence intensity (MFI) data to the transfection efficiency (TE) data shown in Figure 6. (A) MFI data relating to the TE panel 5D. (B) MFI data relating to panel 5D. Each dot represents the mean of an independent triplicate experiment. Mean ± SD (N = 3), *p < 0.05, **p < 0.01, ***p < 0.001.

1. Steffen Honrath. Biorender Publication License Closing the Gap. *Created in BioRender. Honrath, S. (2025) https://BioRender.com/ e57n766* (2025).
